# Sprouty4 negatively regulates ERK/MAPK signaling and the transition from *in situ* to invasive breast ductal carcinoma

**DOI:** 10.1371/journal.pone.0252314

**Published:** 2021-05-28

**Authors:** Ethan J. Brock, Ryan M. Jackson, Julie L. Boerner, Quanwen Li, Meredith A. Tennis, Bonnie F. Sloane, Raymond R. Mattingly

**Affiliations:** 1 Department of Oncology, Wayne State University School of Medicine, Detroit, MI, United states of America; 2 Department of Pharmacology, Wayne State University School of Medicine, Detroit, MI, United states of America; 3 Division of Pulmonary Sciences and Critical Care Medicine, Department of Medicine, University of Colorado Denver Anschutz Medical Campus, Aurora, CO, United states of America; University of Alabama at Birmingham, UNITED STATES

## Abstract

Breast ductal carcinoma *in situ* (DCIS) is a non-obligate precursor of invasive ductal carcinoma (IDC). It is still unclear which DCIS will become invasive and which will remain indolent. Patients often receive surgery and radiotherapy, but this early intervention has not produced substantial decreases in late-stage disease. Sprouty proteins are important regulators of ERK/MAPK signaling and have been studied in various cancers. We hypothesized that Sprouty4 is an endogenous inhibitor of ERK/MAPK signaling and that its loss/reduced expression is a mechanism by which DCIS lesions progress toward IDC, including triple-negative disease. Using immunohistochemistry, we found reduced Sprouty4 expression in IDC patient samples compared to DCIS, and that ERK/MAPK phosphorylation had an inverse relationship to Sprouty4 expression. These observations were reproduced using a 3D culture model of disease progression. Knockdown of Sprouty4 in MCF10.DCIS cells increased ERK/MAPK phosphorylation as well as their invasive capability, while overexpression of Sprouty4 in MCF10.CA1d IDC cells reduced ERK/MAPK phosphorylation, invasion, and the aggressive phenotype exhibited by these cells. Immunofluorescence experiments revealed reorganization of the actin cytoskeleton and relocation of E-cadherin back to the cell surface, consistent with the restoration of adherens junctions. To determine whether these effects were due to changes in ERK/MAPK signaling, MEK1/2 was pharmacologically inhibited in IDC cells. Nanomolar concentrations of MEK162/binimetinib restored an epithelial-like phenotype and reduced pericellular proteolysis, similar to Sprouty4 overexpression. From these data we conclude that Sprouty4 acts to control ERK/MAPK signaling in DCIS, thus limiting the progression of these premalignant breast lesions.

## Introduction

Breast cancer is one of the most common malignancies affecting U.S. women, with up to 25% of these cases being *in situ* disease [1–4]. Breast ductal carcinoma *in situ* (DCIS) is a pre-invasive condition in which abnormal cells proliferate inside the mammary duct [5]. When these cells escape this confined area by invading into the surrounding tissue the lesion is classified as invasive breast ductal carcinoma (IDC), and patient prognosis becomes much less favorable [5, 6]. It is still unclear which DCIS will become invasive and which will remain indolent [7, 8]. As a result, many of these lesions are treated via breast conservation surgery and radiotherapy (with or without hormone therapy) though bilateral mastectomy can be implemented where genetically indicated [1, 8, 9]. Under this standard, DCIS that could have been managed with less aggressive therapy is overtreated, resulting in unnecessary patient risk and financial burden. This dilemma has generated a need for reliable biomarkers or molecular determinants to assess invasive potential [5, 6, 8, 10–13].

There are multiple subtypes of IDC, but treatment options are the most limited for those classified as triple-negative breast cancer (TNBC). TNBCs lack the conventional markers for targeted therapy: expression of estrogen receptor (ER), progesterone receptor (PR), and overexpression of human epidermal growth factor receptor-2 (HER-2) [14]. This leaves chemotherapy as the primary treatment for these patients, though their cancer often responds poorly. One explanation for this comes from a molecular characterization of TNBC subgroups that responded to different therapies [15]. DCIS can also be triple-negative, though few cases are observed in the clinic. This is thought to be a result of their aggressive nature and shorter time to progression [16]. TNBC is more strongly associated with distant recurrence, metastasis, and death when compared to other types of IDC, and the need for a better understanding of disease progression is well recognized [17]. With the advent of better detection systems (such as screening mammography and in some cases MRI), more breast lesions are being detected annually [2, 9]. While early detection is important, reports show that the current treatment standard has room for improvement and a more nuanced understanding of DCIS biology is required for effective disease intervention [10, 18–20].

Our lab has examined the networks and pathways relevant to *in situ* breast disease using cell lines grown in three-dimensional (3D) reconstituted basement membrane (rBM) overlay cultures and next generation/deep sequencing (NGS) [21]. Briefly, of the 157 differentially expressed genes identified by microarray, 63 were found to be up-regulated in the DCIS models using NGS. Further examination using the Genomatix Pathway System identified a highly-enriched common framework (336-fold) in the promoters of three genes throughout the human genome. One of these, *RAP1GAP* has been shown to be a potential switch for progression toward an invasive phenotype [22]. The confirmation of this hit led to further investigation of an equally strong candidate, *SPRY4*, which encodes the protein Sprouty4. Sprouty4 is a human homolog of Sprouty in *Drosophila*, a developmental protein that regulates branching during organogenesis [23, 24]. Sprouty has been shown to inhibit Ras pathway signaling intracellularly by translocating to the plasma membrane in response to growth factor signaling [25]. This change is thought to be important for its role in signal modulation and allows the protein to affect cellular processes such as proliferation, differentiation, motility, and survival [26]. There are four mammalian homologs of Sprouty, designated Sproutys 1–4 [27]. Despite their sequence similarity, the Sprouty homologs lack any recognizable protein interaction domain and do not appear to share the same mechanism of action [28]. Sprouty homologs are known to function differently in various tissue types, and this may be partially accounted for by the variability present in their N-termini [26]. For example, suppression of Sprouty1 was recently found to reduce proliferative signaling in MDA-MB-231 TNBC cells whereas Sprouty4 knockdown increased their proliferative signaling and stem-like properties [29, 30]. Additionally, knockdown of Sprouty2 in human embryonic stem cells was shown to result in decreased proliferation and increased cell death whereas Sprouty4 knockdown produced the opposite effect [31].

Sprouty4 is known to modulate extracellular signal-regulated kinase/mitogen-activated protein kinase (ERK/MAPK) signaling stimulated by receptor tyrosine kinase (RTK) activity [26, 32, 33]. Canonically, the RTK-Ras-ERK pathway is a signaling cascade that governs cellular proliferation, invasion, and survival [34, 35]. GTP-Ras signaling leads to the activation of Raf, MEK1/2, and eventually ERK1/2 which then translocates to the nucleus to regulate gene expression [34–36]. Sprouty proteins have been studied in a number of malignancies but the prevalent activation of ERK/MAPK signaling in breast cancer suggests that they may be particularly relevant to this disease [26, 33, 36–38]. Given that oncogenes often become active earlier in cancer development, it seems reasonable that more delayed molecular changes, such as the loss of growth suppressors, would drive premalignant lesions toward invasion. Considering Sprouty4’s biological role, its apparent importance from our NGS screen, the relevance of the ERK/MAPK pathway in breast cancer, and the lack of treatment options for TNBC, the goal of this study was to elucidate Sprouty4’s role in the progression of DCIS to IDC, with an emphasis on triple-negative disease.

## Materials and methods

### Reagents

Trypsin/EDTA solution and penicillin-streptomycin (pen/strep) were obtained from Cellgro (Herndon, VA). Horse serum was purchased from HyClone Laboratories (Logan, UT). Quenched fluorescein-conjugated collagen type IV (DQ-collagen IV) was obtained from Molecular Probes (Eugene, OR). The MEK inhibitor PD184352 was supplied by Pfizer (New York, NY). The MEK inhibitor U0126 and its inactive analog U0124 were purchased from Calbiochem (San Diego, CA). The MEK inhibitor MEK162 was supplied by Novartis Pharma (Basel, Switzerland). 5x siRNA buffer was obtained from GE Dharmacon (Lafayette, CO). Insulin, hydrocortisone, epidermal growth factor, lipofectamine 2000, opti-MEM reduced serum medium, phosphate-buffered saline (PBS), puromycin, Hoechst 33342, 4′,6-diamidino-2-phenylindole (DAPI), vectashield antifade mounting medium, and Alexa Fluor 555 donkey anti-mouse fluorescent secondary antibody (A-31570) were purchased from Thermo Fisher Scientific (Waltham, MA). Enhanced chemiluminescence detection agents and autoradiography film were purchased from Denville Scientific (Holliston, MA). Horseradish peroxidase-conjugated donkey anti-mouse and donkey anti-rabbit IgG antibodies were purchased from Jackson ImmunoResearch Laboratories Inc. (West Grove, PA). Rabbit anti-Sprouty4 (ab115557) antibody was purchased from Abcam (Cambridge, MA). Rabbit anti-Sprouty1 (D9V6P; #13013), rabbit anti-Sprouty2 (D3G1A; #14954), rabbit anti-Slug (C19G7; #9585), rabbit anti-phospho-MEK1/2 Ser217/221 (41G9; #9154), rabbit anti-total MEK1/2 (D1A5; #8727), rabbit anti-phospho ERK1/2 (D13.14.4E; #4370), and rabbit anti-total MAPK (#9102) antibodies were from Cell Signaling (Danvers, MA). Mouse anti-phospho-MAPK (MAPK-YT; #M8159) antibody, fluorescein isothiocyanate labeled phalloidin (P5282), and phorbol 12-myristate 13-acetate, phenylmethylsulfonyl fluoride, leupeptin, and aprotinin were obtained from Sigma-Aldrich (St. Louis, MO). Mouse anti-E-cadherin (#610181) antibody was purchased from BD Biosciences (San Jose, CA). Mouse beta-tubulin (clone E7) antibody was obtained from the Developmental Studies Hybridoma Bank (Iowa City, IA). Reduced growth factor rBM (Cultrex; #3445-005-01) was purchased from Trevigen (Gaithersburg, MD).

### Cell lines and cell culture

The MCF10 human breast epithelial progression series of cells (MCF10A, MCF10.AT1, MCF10.DCIS, and MCF10.CA1d) were obtained from the Biobanking and Correlative Sciences Core at the Karmanos Center Institute, Detroit, MI. Cell lines were authenticated using the STR PowerPlex 16 System (Promega) and confirmed to be free of mycoplasma by microscopy (MycoFluor; Thermo Fisher Scientific) and PCR (Venor GeM; Sigma-Aldrich). All cell lines were maintained as monolayer cultures at 37°C with 5% CO_2_. 2D culture was performed using DMEM/F-12, HEPES, no phenol red medium (Fisher Scientific; 11039047) supplemented with 5% horse serum, 1% pen/strep, 10 μg/mL insulin, 0.5 μg/mL hydrocortisone, and 20 ng/mL epidermal growth factor. For 3D culture where the endpoint was immunoblotting, dishes were coated with 16 mg/mL Cultrex. After solidification of the matrix, a single-cell suspension in 3D assay medium (DMEM/F-12, HEPES, no phenol red medium supplemented with 2% horse serum, 2% Cultrex, 1% pen/strep, 960 ng/mL insulin, 50 ng/mL hydrocortisone, and 0.5 ng/mL epidermal growth factor) was pipetted on top of the matrix and grown for eight days, with assay media being changed after four days. For 3D immunocytochemistry, assay media were not changed during the eight-day period.

### Viral infection for stable overexpression and shRNA knockdown of Sprouty4

Plasmids from bacteria transformed with each of the two pLPCX/Spry4 constructs were extracted and purified using Biorad’s Quantum Prep Plasmid Miniprep Kit (Hercules, CA) [37]. The contents of each construct were verified using Sanger sequencing services provided by Wayne State University. Overexpression of Sprouty4 in the MCF10.CA1d cell line was achieved using HEK293T cells to package 3 μg of plasmid inside retrovirus as previously described [39]. MCF10.CA1d cells were transduced for six hours then allowed to recover before starting negative selection with 5 μg/mL puromycin. Two independent isolates of Sprouty4 with 6x His tags following the C-terminal residues were generated and labeled MCF10.CA1d/S1 and MCF10.CA1d/S2. The transduced Sprouty4 construct migrates at a molecular weight of 37 kDa (compared to 35 kDa for endogenous Sprouty4) on SDS-PAGE. The MCF10.CA1d/S2 cells exhibited a stronger overexpression of Sprouty4 compared to MCF10.CA1d/S1 cells, allowing for the observation of dose-dependent effects.

Stable knockdown of Sprouty4 was achieved in the MCF10.DCIS cell line using two non-overlapping shRNA constructs. Bacterial stocks containing Sprouty4-targeting sequences (#RHS3979-9623883; #RHS3979-9623885) or an empty vector control (#RHS4080) were obtained from GE Dharmacon (Lafayette, CO). Plasmids were extracted and purified using Biorad’s Quantum Prep Plasmid Miniprep Kit (Hercules, CA), and their sequences were verified using Sanger sequencing services provided by Genewiz (South Plainfield, NJ). The antisense construct sequences were as follows: ATAGTTGACCAGAGTCTGGGC for knockdown #1 (#RHS3979-9623883), ATGTGGTCTAAGAGCCGTTGG for knockdown #2 (#RHS3979-9623885), and ACCGGACACTCGAGCACTTTTTGAATTC for the empty vector control (#RHS4080). Neither of the knockdown constructs were found to target *SPRY1* or *SPRY2* using the NCBI Blastn alignment search as well as through manual sequence reading. HEK293T cells were used to package 3.5 μg of construct inside lentivirus as previously described [39]. MCF10.DCIS cells were transduced for six hours then allowed to recover for two days before starting negative selection with 5 μg/mL puromycin. Cultures were maintained in growth medium containing 2.5 μg/mL puromycin.

### siRNA-mediated transient knockdown of Sprouty4 in MCF10.DCIS cells

1x10^5^ cells were seeded in each well of a 6-well plate and allowed to grow for three subsequent days. On day one, cells were transfected with a complex composed of lipofectamine 2000 and 30 nM siRNA (final concentration) obtained from GE Dharmacon (Lafayette, CO). The siRNA target sequences were as follows: GCACGAAUGAGGACGAUGA for *SPRY4* siRNA #1 (#D-015457-01-0002), UGUGGAGAAUGACUACAUA for *SPRY4* siRNA #2 (#D-015457-02-0002), CAACGGCUCUUAGACCACA for *SPRY4* siRNA #3 (#D-015457-03-0002), and UAGCGACUAAACACAUCAA for the non-targeting control siRNA (#D-001210-01-05). None of the constructs were found to target *SPRY1* or *SPRY2* using the NCBI Blastn alignment search as well as through manual sequence reading. Briefly, siRNA and lipofectamine 2000 were each diluted in opti-MEM reduced serum medium, then mixed and allowed to form complexes for 20 minutes. After these complexes were created, 500 μL was mixed with growth medium 1:1 and added to each well. siRNA was allowed to incubate with the cells for four hours before an additional 1mL of growth media was added to each well. Cells were incubated this way for the next 48 hours and then harvested.

### Immunohistochemical staining and analysis of patient tissue samples

Tissue microarrays (TMA) BR8011 (enriched for normal and DCIS tissues), BR487b (enriched for triple-negative or TN IDCs) and BR1504a (enriched for human epidermal growth factor receptor-2 or HER2, and estrogen/progesterone receptor or ER/PR expressing IDCs) were purchased from Biomax (Rockville, MD). Slides were processed for immunohistochemistry (IHC) using optimized protocols and antibodies for Sprouty4 (ab115557) and phosphorylated ERK1/2 (D13.14.4E; #4370). Paraffin sections were de-waxed in a xylene-ethanol series. Endogenous peroxides were removed by 1.2% hydrogen peroxide/methanol incubation at room temperature for 30 minutes. Heat-induced epitope retrieval was performed with a pH 6 citrate buffer in a BIOCARE Decloaking Chamber. A one-hour blocking step with 10% goat serum in PBS was done prior to adding primary antibody overnight. Detection was performed using Life Technologies Broad Spectrum 3,3’-diaminobenzidine SuperPicTure Polymer Detection Kit (#879663), and counterstained with Mayer’s Hematoxylin. Sections were then dehydrated through a series of ethanol to xylene washes and cover slipped with Permount (Thermo Fisher Scientific). Indica Labs’ TMA software module (Corrales, NM) was used to segment the tissue spots on the slide and measure Sprouty4 and phosphorylated ERK1/2 (pERK1/2) staining. The staining for pERK1/2 was then classified as percent negative, weak, moderate or strongly positive, taking the entire analysis region into consideration. Parameters for weak, moderate, and strongly positive staining were set manually by an experienced investigator, using positive and negative control slides as a gating reference prior to analysis of the TMAs. The staining classification was defined by the software as the percent of positive cells in the total cells counted. The total number of cells was determined by counting counterstained nuclei. Percent positive stain of Sprouty4 was also analyzed. Images of representative tissue spots were taken at 20x magnification using a Zeiss Axiovert 200 light microscope.

### Immunocytochemistry

#### 2D cultures

Immunocytochemistry was performed as previously described [40] with the following exceptions: Blocking was performed with 3% BSA/PBS for two hours at room temperature, followed by incubation with primary anti-E-cadherin antibody (1:500) overnight at 4°C. The next day, Alexa Fluor 555 donkey anti-mouse fluorescent secondary antibody and fluorescein isothiocyanate labeled phalloidin were each diluted 1:500 in 3% BSA/PBS and applied for two hours at room temperature. DAPI was subsequently diluted 1:1000 in PBS and applied to the chambers for 10 minutes. Following this, the samples were washed 5x for five minutes each with PBS and then aspirated dry from one corner. Coverslips were mounted using vectamount antifade solution and cells were imaged on a LSM 780 confocal microscope (Carl Zeiss Microscopy, Thornwood, NY, USA) using a 63x objective.

#### 3D cultures

100 μL of 16 mg/mL Cultrex was administered to the center of each 35 mm dish and incubated at 37°C for 30 minutes to accelerate solidification of the matrix. Simultaneously, a single-cell suspension in assay medium containing 8000 cells/mL was generated. 400 cells were then seeded on top of the matrix and allowed to attach for 45 minutes. Two milliliters of assay medium were then added to each dish and cultured for eight days. Once ready, each dish was aspirated and subjected to the following: rinsed once with warm PBS; fixed with cold 4% paraformaldehyde for 20 minutes; rinsed again with warm PBS; permeabilized with cold 0.2% Triton X-100/PBS for 10 minutes; quenched 3x for five minutes with 0.75% Glycine/PBS; rinsed twice with warm PBS; blocked with 3% BSA/PBS for one hour at room temperature; incubated with primary anti-E-cadherin antibody (1:500) overnight at 4°C. The next day each culture was washed 3x for 10 minutes with warm PBS, then Alexa Fluor 555 donkey anti-mouse fluorescent secondary antibody and fluorescein isothiocyanate labeled phalloidin were each diluted 1:500 in 3% BSA/PBS along with DAPI (1:1000) and applied for two hours at room temperature. Finally, three 10 minute washes with warm PBS were performed. Images were collected with a Zeiss LSM 510 Meta nonlinear optical confocal microscope (Carl Zeiss Microscopy, Thornwood, NY, USA) using a 63x water immersion objective. 3D reconstructions of optical sections were generated using Volocity software v.6.3.1 as described previously [41].

### Live cell proteolysis assay

Live cell proteolysis was studied as previously described [41] with the following exceptions: 100 μL of Cultrex containing 25 μg/mL dye-quenched (DQ)-collagen IV were incubated for 30 min at 37°C to accelerate solidification of the matrix. During this time a single-cell suspension containing 5000 cells/mL of assay medium was generated, and 250 cells were seeded on top of the matrix (once retrieved) and allowed to attach for 45 minutes. Two milliliters of assay medium were then added to each dish and cultured for eight days. Drugs were administered 48 hours before imaging on a LSM 780 confocal microscope (Carl Zeiss Microscopy, Thornwood, NY, USA) equipped with a 40x water immersion objective and controlled environmental chamber that maintains a 5% CO_2_/humidified atmosphere at 37°C. Cell nuclei were stained using Hoechst 33342 and proteolysis was determined by the generation of green fluorescent degradation products. 3D reconstructions of optical sections were generated and the amount of proteolytic degradation per cell was quantified using Volocity software v.6.3.1 as described previously [41]. *En face* images of 3D reconstructed optical sections were taken to show fluorescent cleavage products. Data were collected from three independent experiments performed in triplicate.

### Immunoblotting

#### 2D cultures

Immunoblotting was performed as previously described [40] with the following exceptions: SDS-PAGE using minigels was executed for ~90 minutes at constant voltage (100V) before minigels were transferred overnight at 30V. After blocking solution was removed and the membrane rinsed with TBS-T, primary antibody was incubated overnight at 4°C. The following day after removal of the primary antibody and washing steps were complete, secondary antibody was incubated with the membrane for one hour then washed 2x for 10 minutes with TBS-T.

#### 3D cultures

5x10^5^ cells were seeded and grown for eight days in culture. Media were aspirated and cultures were stored on ice. Each dish was briefly rinsed with PBS, then lysates were collected in the presence of a PBS-EDTA/inhibitor solution (PBS-EDTA 5 mM, pH 7.4; 50 mM Na₃VO₄; 500 mM NaF; 25 μg/mL leupeptin; 25 μg/mL aprotinin; 174 μg/mL phenylmethylsulfonyl fluoride). Lysates were centrifuged at 4°C and 900g for three minutes then the remaining solution was aspirated. RIPA buffer supplemented with the same inhibitors as described above was then added. Lysates were resuspended, subjected to brief sonication, then mixed with 2x Laemmli buffer (described here [40]) and heated at 100°C for 5 minutes. Because protein concentrations could not be used to standardize the lysates (due to the presence of the matrix), the lysates were initially loaded based on volume and tested for content of tubulin by immunoblotting. If necessary, loading adjustments were made to equalize the tubulin contents of the samples. SDS-PAGE and immunoblotting were carried out as described above for 2D detection.

### Invasion assays

6x10^5^ pLKO.1 MCF10.DCIS control, pLKO.1 MCF10.DCIS Sprouty4 knockdown, MCF10.CA1d, as well as MCF10.CA1d/S1 and MCF10.CA1d/S2 Sprouty4 overexpression cell lines were seeded in serum-free media on BD cell culture inserts (8 μm pore size; Franklin Lakes, NJ) pre-coated with 1.5 mg/mL Cultrex. Inserts were placed in a 24-well plate and cells were allowed to invade toward serum-containing growth media for 24 hours. After this period, cells that did not invade were removed using cotton tipped applicators and each filter (containing the invasive cells) was stained using the Kwik-Diff stain kit (Thermo Fisher Scientific) per the manufacturer’s instructions. Filters were mounted and subsequently visualized using a Zeiss Axiovert 200 light microscope (Carl Zeiss Microscopy, Thornwood, NY, USA). Invading cells were counted by ImageJ software (NIH) with the help of two blinded individuals. Data for all cell lines were collected from three independent experiments with a minimum of three technical replicates per condition.

## Results

### Sprouty4 expression and ERK1/2 phosphorylation are inversely related in human patient tissues

Our previous work has examined networks and pathways relevant to *in situ* breast disease [21]. To do this we used transcriptomic analyses to compare the expression profiles of three DCIS cell lines to a non-transformed breast epithelial cell line. Cells were grown in 3D rBM overlay cultures because research has shown that the behavior of cancer cells in 3D matrices is more reflective of an *in vivo* response when exposed to drugs and radiotherapy than if they are cultured on plastic [42–46]. Bioinformatics performed on these data identified multiple promising candidate genes. We showed that the reduced expression of one such candidate, *RAP1GAP*, is a potential switch for progression toward an invasive phenotype [22]. Another candidate from this screen was *SPRY4*, which encodes the protein Sprouty4. Analysis of IDC vs. normal breast tissue using the Oncomine database supports this finding, as *SPRY4* transcript was significantly underexpressed in IDC samples in both the TCGA (p = 1.06x10^-5^) and Curtis Breast (p = 8.78x10^-10^) datasets (see S1 Fig and S2 Fig, respectively). Additionally, IHC data from The Human Protein Atlas (https://www.proteinatlas.org/ENSG00000187678-SPRY4/tissue) comparing normal and cancerous tissue expression revealed Sprouty4 to be lower in the majority of breast cancer samples when compared to normal tissue (7 of 12). Taken together, these datasets suggest the importance of Sprouty4 in DCIS and prompted further investigation.

**Fig 1:**
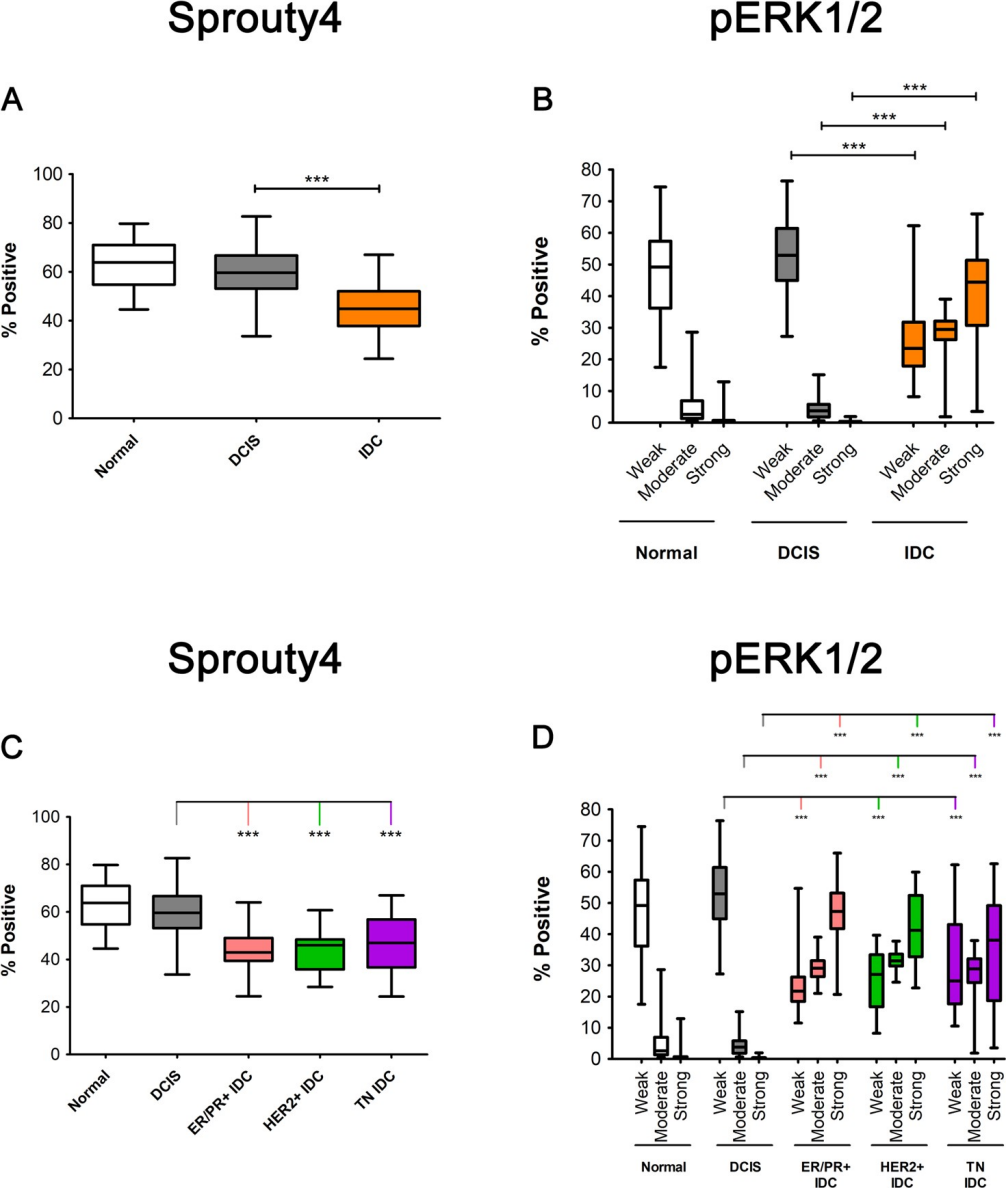
Sprouty4 levels are significantly reduced in human IDCs relative to normal and DCIS tissues whereas phosphorylated ERK1/2 shows the opposite pattern. Human tissue microarrays (TMAs) containing samples of normal breast tissue adjacent to tumor, n = 24; DCIS, n = 45; and IDC, n = 169; were processed for IHC using optimized protocols and antibodies for Sprouty4 and phosphorylated ERK1/2 (pERK1/2). (A, C) One-way ANOVA employing Tukey’s multiple comparison test showed significant differences in the percentage of cells that were positive for cytosolic Sprouty4 expression when comparing normal or DCIS tissue to IDC that was pooled or separated by subtype (α = 0.001, 99.9% CI; *** p < 0.001). Whiskers represent the minimum and maximum values and bars represent the median. (B, D) A Kruskal-Wallis test employing Dunn’s test of multiple comparisons showed significant differences in the percentage of cells that were positive for pERK1/2 expression when comparing normal or DCIS tissue to IDC that was pooled or separated by subtype (α = 0.001, 99.9% CI; *** p < 0.001).

To evaluate Sprouty4’s protein expression in the context of breast cancer progression, we stained human TMAs containing samples of tumor-adjacent normal breast (n = 24), DCIS (n = 45), and IDC (n = 169) using IHC. Expression was significantly (p < 0.001) reduced in IDC samples compared to DCIS or normal tissues (see Fig 1A; S3 Fig). Sprouty4’s reported ability to regulate ERK/MAPK signaling prompted staining for phosphorylated ERK1/2. Significantly more cells exhibited weak ERK1/2 phosphorylation in normal and DCIS tissues whereas more cells exhibited moderate and strong ERK/MAPK phosphorylation in IDC samples (p < 0.001) (see Fig 1B; S3 Fig). This inverse relationship between Sprouty4 expression and phosphorylated ERK1/2 signaling was observed both when IDC sample data were pooled (see Fig 1A and 1B) as well as when they were separated into ER/PR+ (n = 67), HER2+ (n = 22), and TNBC (n = 80) breast cancer subtypes (see Fig 1C and 1D).

### Sprouty4 is highly expressed at the DCIS stage and reduced with transition to IDC in the MCF10 progression series

**Fig 2:**
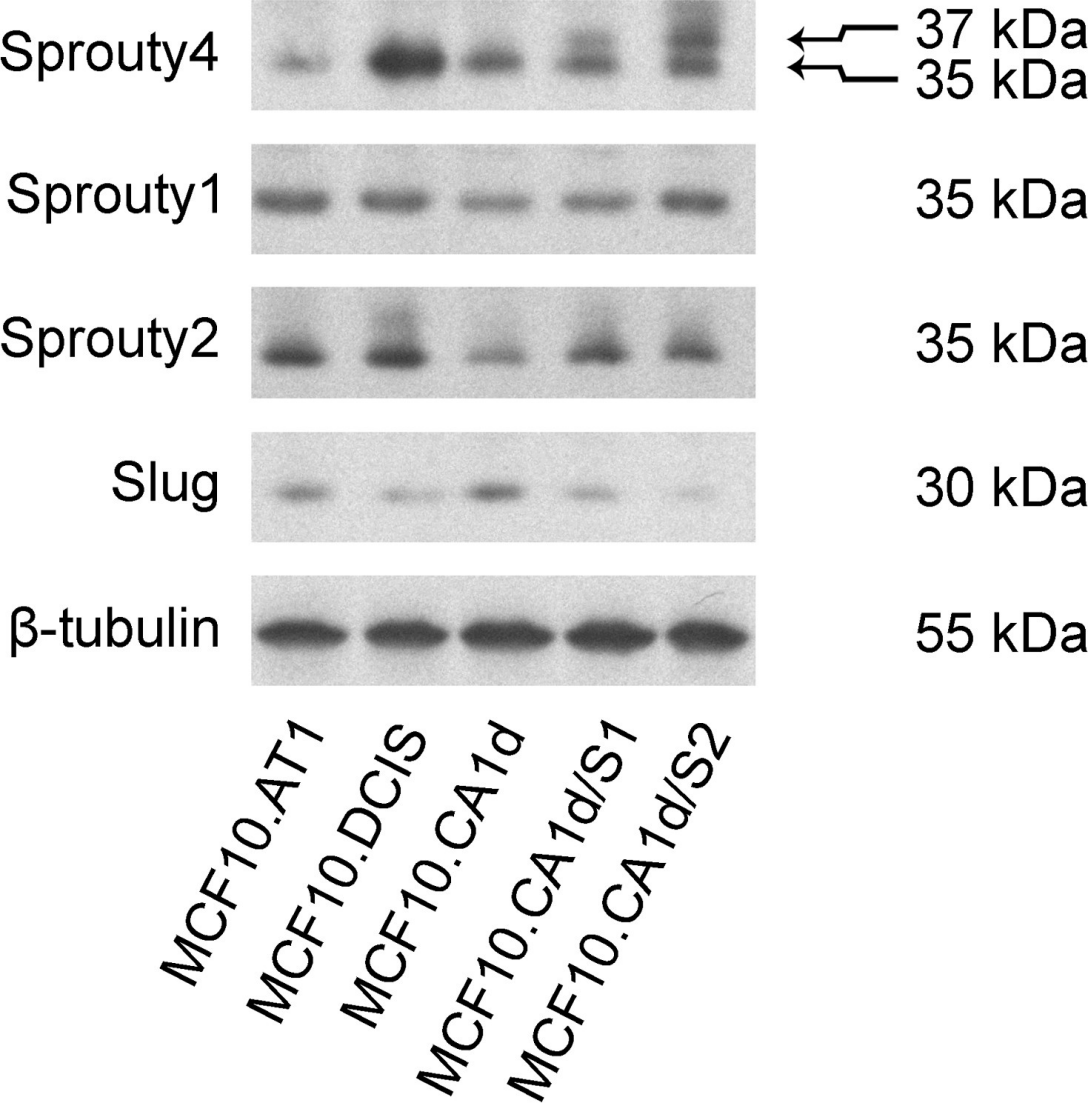
Sprouty4 is highly expressed at the DCIS stage of the MCF10 progression series. The progression series represents triple-negative models of atypical hyperplasia (MCF10.AT1), ductal carcinoma *in situ* (MCF10.DCIS), and invasive ductal carcinoma (MCF10.CA1d). MCF10.CA1d/S1 (S1) and MCF10.CA1d/S2 (S2) are two independent retroviral overexpressions of Sprouty4 in MCF10.CA1d cells where the transduced Sprouty4 construct migrates at a molecular weight of 37 kDa compared to 35 kDa for endogenous Sprouty4. Cultures were grown in 3D rBM overlay conditions for eight days. Membranes were immunoblotted for Sprouty4, Sprouty1, Sprouty2, Slug, and β-tubulin. Expression levels are representative of three independent experiments.

To define the role of Sprouty4 in DCIS and IDC we interrogated protein levels via immunoblotting of 3D culture lysates using the MCF10 series as a model of breast cancer progression. This series of isogenic cell lines recapitulates the various stages of progression [47–53], and includes non-transformed breast epithelial cells (MCF10A), premalignant variants (MCF10AT and MCF10DCIS), and malignant variants (MCF10CA) [4, 50–53]. We tested whether this *in vitro* system reiterated the pattern of Sprouty4 expression that we observed by IHC in patient tissue. The results showed the highest Sprouty4 expression in the premalignant MCF10.DCIS cell line and lower Sprouty4 expression in the invasive MCF10.CA1d cell line (see Fig 2). To check whether this expression pattern was specific to Sprouty4, both Sprouty1 and Sprouty2 were also examined. Sprouty1 and 2 levels were broadly comparable across the models, with a trend toward a decrease in invasive disease.

To investigate the importance of Sprouty4 in disease progression, we created two independent retroviral overexpressions of Sprouty4 (MCF10.CA1d/S1 and MCF10.CA1d/S2) and determined the effect this had on activation of the ERK/MAPK pathway. In breast cancer cells prolonged pharmacological inhibition (i.e., 48 hours) of the ERK/MAPK pathway decreases protein expression of Slug [54]. Slug is a transcription factor whose expression is downstream of the ERK/MAPK cascade [55, 56]. These reports suggest that Slug can be used as a surrogate marker for chronic ERK1/2 activity in 3D culture. We found that Slug levels decreased with Sprouty4 overexpression (see Fig 2). Cell lines with stronger Sprouty4 expression (i.e., MCF10.DCIS, MCF10.CA1d/S1 and MCF10.CA1d/S2) exhibited lower levels of Slug, suggesting that active ERK1/2 signaling was also lower (see Fig 2). This conclusion aligns with our IHC data, where Sprouty4 expression was inversely related to ERK1/2 phosphorylation.

### Modulation of Sprouty4 expression regulates ERK1/2 phosphorylation in cellular models of IDC

**Fig 3:**
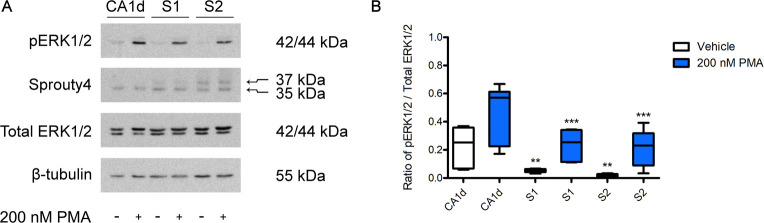
Overexpression of Sprouty4 in MCF10.CA1d cells suppresses ERK/MAPK phosphorylation. (A) MCF10.CA1d control and Sprouty4 overexpressing cells (MCF10.CA1d/S1 and MCF10.CA1d/S2) grown in 2D culture were serum starved for 24 hours, then treated with DMSO (vehicle) or 200 nM phorbol 12-myristate 13-acetate for 10 minutes. Membranes were immunoblotted for Sprouty4, phosphorylated ERK1/2 (pERK1/2) as well as total ERK1/2, and β-tubulin. Expression levels are representative of three independent experiments. (B) ERK1/2 phosphorylation was quantified using densitometry and plotted using Graphpad Prism. One-way ANOVA employing a repeated measures test and Bonferroni correction was used to assess significance (α = 0.01, 99% CI). Whiskers represent the minimum and maximum values and bars represent the median. Differences were observed under both unstimulated (** p < 0.01) and stimulated (*** p < 0.001) conditions.

To more directly characterize whether increasing Sprouty4 led to a decrease in ERK1/2 signaling, we performed an acute stimulation experiment. MCF10.CA1d as well as MCF10.CA1d/S1 and MCF10.CA1d/S2 cells were challenged with phorbol ester for 10 minutes. Both the MCF10.CA1d/S1 and MCF10.CA1d/S2 overexpression lines (here S1 and S2) exhibited significantly lower levels of phosphorylated ERK/MAPK under both stimulated (p < 0.001) and unstimulated (p < 0.01) conditions compared to control (see Fig 3).

**Fig 4:**
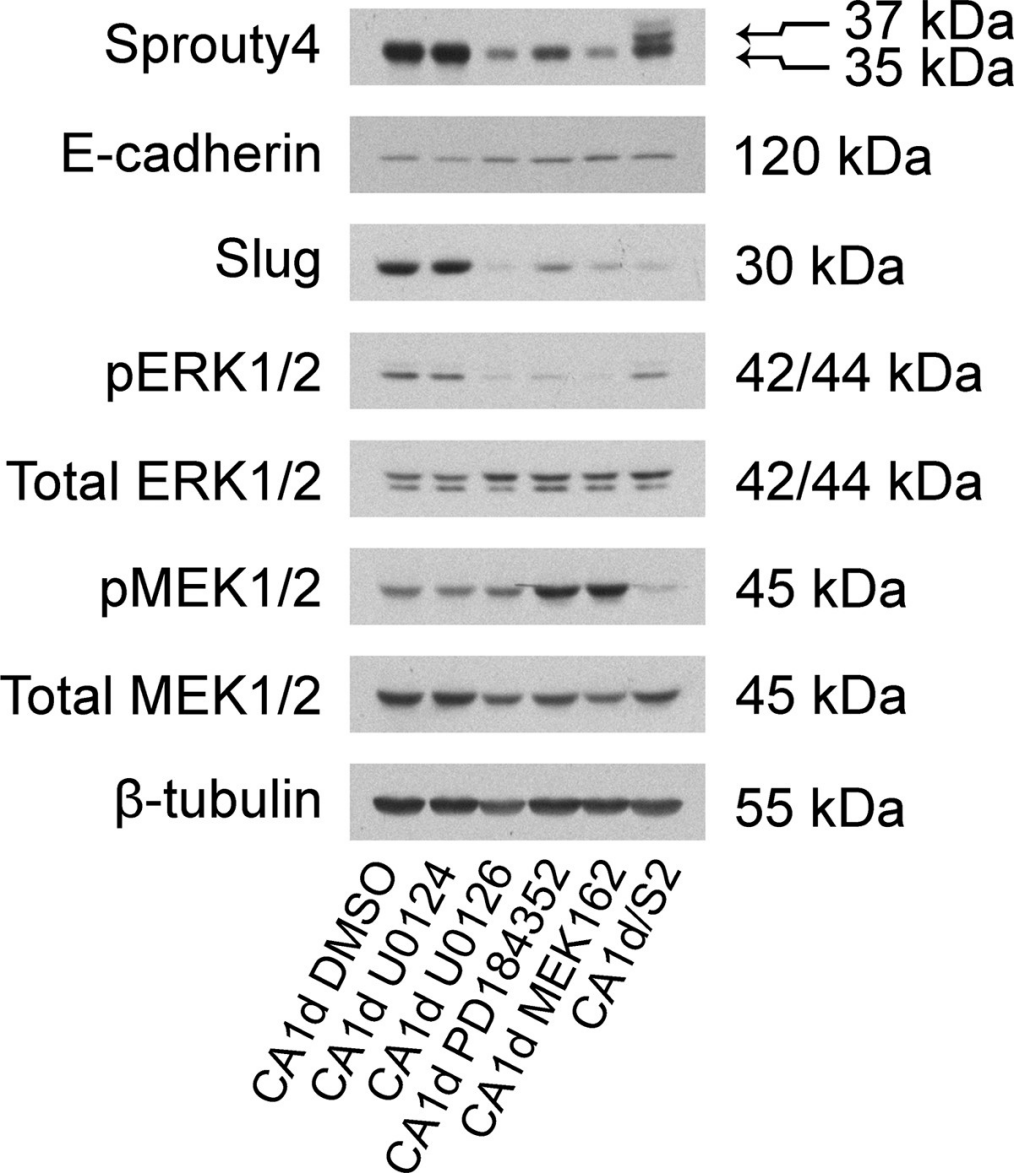
Sprouty4 regulates and responds to the ERK/MAPK pathway. The ERK/MAPK pathway was targeted in MCF10.CA1d cells cultured in 2D using three separate MEK1/2 inhibitors: U0126 (10 μM), PD184352 (100 nM) and MEK162 (100 nM) for 48 hours. U0124 (10 μM) is a negative control for U0126. Membranes were immunoblotted for Sprouty4, E-cadherin, Slug, phosphorylated ERK1/2 (pERK1/2), phosphorylated MEK1/2 (pMEK1/2), total ERK1/2, total MEK1/2, and β-tubulin. Expression levels are representative of three independent experiments.

To better understand how Sprouty4 specifically regulates and responds to ERK/MAPK signaling, we inhibited MEK1/2 in MCF10.CA1d cells grown in 2D culture for 48 hours using three different compounds (U0126, 10 μM; PD184352/CI-1040, 100 nM; MEK162/binimetinib, 100 nM). We interrogated the effects of these allosteric inhibitors on phosphorylated ERK1/2, phosphorylated MEK1/2, Sprouty4, and Slug in control and Sprouty4 overexpressing cells (see Fig 4). Treatment with vehicle or U0124 (a negative control for U0126) showed relatively high ERK1/2 phosphorylation and Slug expression. When MEK inhibitors were administered or Sprouty4 was overexpressed, ERK1/2 phosphorylation and Slug expression were reduced. The decrease in ERK1/2 signaling induced by MEK inhibitors was also accompanied by a reduction in Sprouty4 levels. Importantly, when MCF10.CA1d cells were treated with PD184352 and MEK162 a feedback loop could be seen, resulting in increased levels of phosphorylated MEK1/2. With Sprouty4 overexpression the opposite is true, suggesting that Sprouty4 acts upstream of MEK1/2. This is consistent with results showing that Sprouty4 acts upstream or at the level of Ras or with Raf proteins [24, 57, 58].

### Sprouty4 overexpression induces phenotypic reversion in MCF10.CA1d cells

**Fig 5:**
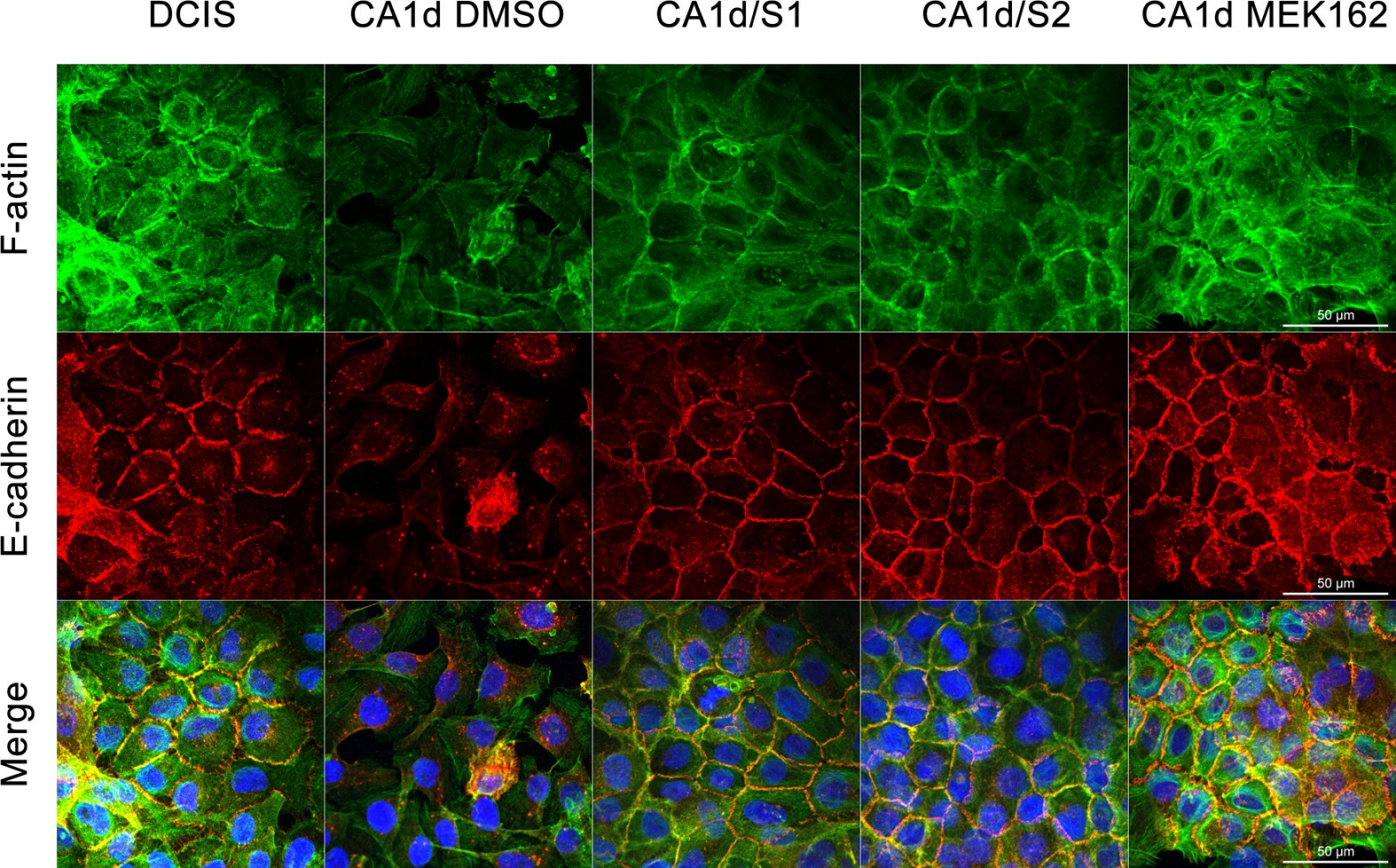
Sprouty4 overexpression promotes an epithelial-like phenotype. MCF10.DCIS, MCF10.CA1d control and Sprouty4 overexpressing cell lines (CA1d/S1 and CA1d/S2) were grown in 2D on glass coverslips for four days. During the last 48 hours CA1d cells were treated with either DMSO or 100 nM MEK162. Confocal fluorescent imaging was performed using a 63x objective. Images show phalloidin-FITC for filamentous actin (green), indirect immunofluorescence for E-cadherin (red), and DAPI for nuclei (blue); size bar = 50 μm. Sprouty4 overexpression and MEK inhibition lead to the formation of actin cortical ring-like structures, and a change in E-cadherin localization that, together, resemble an epithelial phenotype. Images are representative of three independent experiments.

A minimal increase in E-cadherin expression was also observed in MCF10.CA1d cells treated with MEK inhibitors or overexpressing Sprouty4 (see Fig 4). We hypothesized that this may be a consequence of removing upstream inhibition, as Slug is a transcriptional repressor of E-cadherin in breast carcinoma cell lines [59]. To identify any changes in E-cadherin localization, immunofluorescence for E-cadherin and filamentous actin was performed (see Fig 5). MCF10.DCIS, MCF10.CA1d, and MCF10.CA1d cells overexpressing Sprouty4 (CA1d/S1 and CA1d/S2) were grown in 2D on glass coverslips for four days. During the last 48 hours, MCF10.CA1d cells were treated with either DMSO vehicle control or 100 nM MEK162. MCF10.DCIS cells exhibited relatively intact cell-cell junctions with E-cadherin localized to the cell surface and cortical actin rings (characteristic of epithelial cells). In invasive MCF10.CA1d cells, a dramatic reorganization of the actin cytoskeleton was visible, and E-cadherin staining was not localized at the cell surface. However, when Sprouty4 was overexpressed or the ERK/MAPK pathway was pharmacologically inhibited in MCF10.CA1d cells, E-cadherin re-localized to the cell surface and there was reorganization of the actin cytoskeleton (see Fig 5).

**Fig 6:**
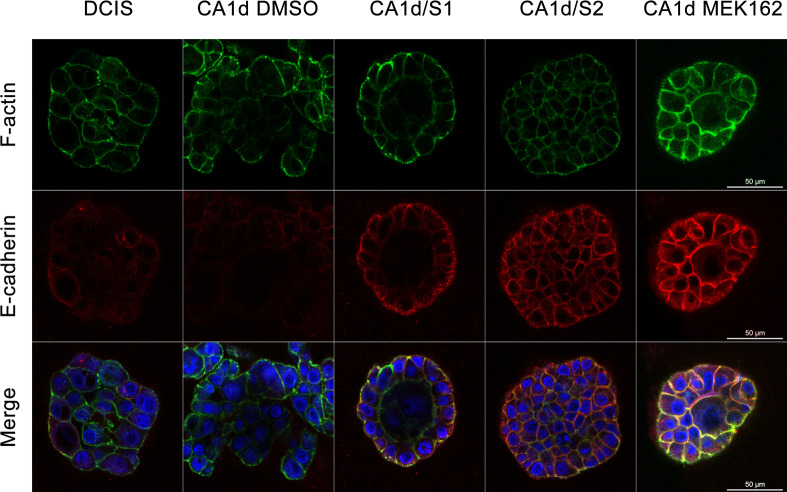
Sprouty4 overexpression supports the restoration of E-cadherin. MCF10.DCIS, as well as MCF10.CA1d control and Sprouty4 overexpressing cell lines (CA1d/S1 and CA1d/S2) were grown in 3D rBM overlay cultures for eight days. During the last 48 hours, CA1d cells were treated with either DMSO or 100 nM MEK162. Confocal fluorescent imaging was performed using a 63x objective. Equatorial planes of 3D reconstructions show phalloidin-FITC for filamentous actin (green), indirect immunofluorescence for E-cadherin (red), and DAPI for nuclei (blue); size bar = 50 μm. The localization of E-cadherin staining in the Sprouty4 overexpressing and MEK inhibited cells is consistent with relocation to the cell surface and the restoration of adherens junctions. Images are representative of three independent experiments.

**Fig 7:**
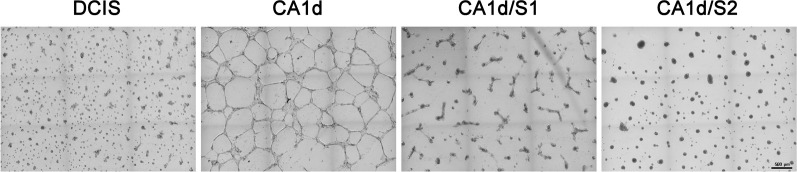
Sprouty4 overexpression induces phenotypic reversion of MCF10.CA1d cells. MCF10.DCIS, MCF10.CA1d control and Sprouty4 overexpressing cell lines (MCF10.CA1d/S1 and MCF10.CA1d/S2) were grown in 3D rBM overlay cultures. By 48 hours, structural differences were observed between the MCF10.CA1d and overexpression lines. Sprouty4 overexpressing cells exhibited phenotypic reversion and resembled the isogenic DCIS line more than the parental invasive line. Differential interference contrast images were captured on a Zeiss Cell Observer spinning disk confocal microscope using a 5x objective and stitching nine contiguous fields (3x3) together to ensure reproducibility; size bar = 500 μm. Images are representative of three independent experiments.

We further investigated the effects of Sprouty4 overexpression and MEK inhibitor treatment using rBM overlay cultures. 3D structures showed changes in E-cadherin localization as well as restructuring of the cellular architecture consistent with effects observed in 2D cultures (see Fig 6; S1–S5 Videos). The partial formation of acinar-like structures was also observed with Sprouty4 overexpression (CA1d/S1 and CA1d/S2) as well as MEK inhibition (CA1d MEK162), suggesting these modifications are able to induce phenotypic reversion (i.e., from invasive structures to more organized structures). In differential interference contrast images, the phenotypic differences between control MCF10.CA1d cells and experimental MCF10.CA1d cells that have either been treated with a MEK inhibitor in 2D culture (see S4 Fig) or overexpress Sprouty4 in 3D culture (see Fig 7) were striking. When levels of Sprouty4 expression were high, the 3D cellular structures more closely resembled the spheroids seen with MCF10.DCIS than the parental MCF10.CA1d cell line (see Fig 7). Together, these data indicate that changes in E-cadherin expression and localization, as well as the restructuring of the actin cytoskeleton due to Sprouty4 expression, ultimately occur as a result of regulation of the ERK/MAPK pathway.

### Overexpression of Sprouty4 negatively regulates cellular proteolysis and invasion whereas knockdown increases ERK signaling and promotes invasion

**Fig 8:**
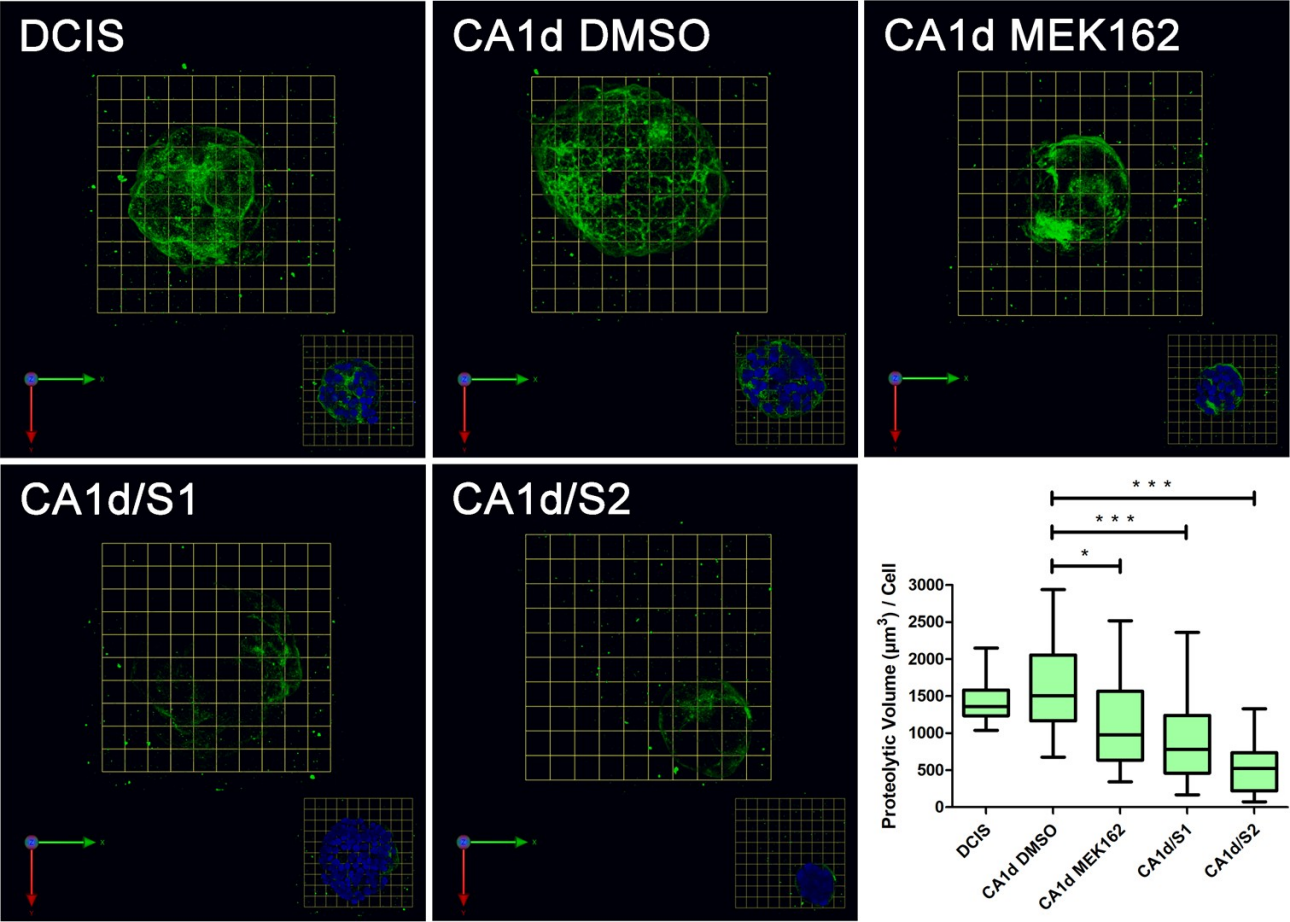
MCF10.CA1d cells exhibit an aggressive phenotype that can be reduced by MEK inhibition and overexpression of Sprouty4. MCF10.DCIS, MCF10.CA1d control and Sprouty4 overexpressing cells were grown in 3D rBM overlay cultures containing dye-quenched collagen IV for eight days, then imaged live. During the last 48 hours, CA1d cells were treated with either DMSO or 100 nM MEK162. Collagen-IV degradation products (green) are shown in en face views of 3D reconstructions of optical sections; 1 grid unit = 21 μm. The volume of degradation products was quantified per cell using Volocity and plotted using Graphpad Prism. One-way ANOVA employing Bonferroni’s correction was used to assess significance (α = 0.05, 95% CI). Whiskers represent the minimum and maximum values and bars represent the median. Statistically significant decreases in proteolytic activity were observed upon MEK inhibition and Sprouty4 overexpression (* p < 0.05; *** p < 0.001). Data were collected from three independent experiments.

**Fig 9:**
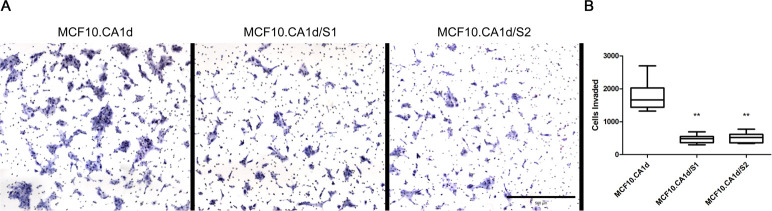
Stable overexpression of Sprouty4 in MCF10.CA1d cells reduces invasion. (A) MCF10.CA1d control and Sprouty4 overexpressing cells (MCF10.CA1d/S1 and MCF10.CA1d/S2) were serum-starved overnight and allowed to invade though 1.5 mg/mL of matrix for 24 hours via Boyden chamber assay. Representative images of cellular invasion (scale bar = 500 μm). (B) Invasion was quantified and plotted using GraphPad Prism. A Kruskal-Wallis test employing Dunn’s test of multiple comparisons showed significant differences in invasion when comparing Sprouty4 overexpression lines to control (α = 0.01, 99% CI; ** p < 0.01). Whiskers represent the minimum and maximum values and bars represent the median. Data were collected from three independent experiments.

In addition to governing proliferation and survival, the ERK/MAPK pathway is known to promote invasion [60, 61]. Interestingly, preventing ERK activation in cancer cells has led to decreased gene expression and enzymatic activity of MMP-9 [60, 62]. This proteinase has also been linked to extracellular proteolysis and was shown to be modulated by Sprouty4 expression in lung cancer [37]. These findings, our data, as well as the importance of the ERK/MAPK pathway in breast cancer led us to ask if Sprouty4 expression was sufficient to alter the invasive phenotype of breast cancer cells *in vitro*. To answer this, we performed live cell proteolysis assays as well as Boyden chamber invasion assays. These live cell proteolysis assays assessed the invasive characteristics of multi-cellular structures via their ability to cleave quenched fluorescent proteins mixed into the surrounding stroma, in this case dye-quenched (DQ)-collagen IV mixed into rBM [41]. MCF10.DCIS, MCF10.CA1d/S1, MCF10.CA1d/S2, and MCF10.CA1d cells treated for 48 hours with either DMSO vehicle control or 100 nM MEK162 were grown in 3D culture for eight days and DQ-collagen IV cleavage products were quantified on a per cell basis (see Fig 8). Significant decreases in DQ-collagen IV proteolysis were observed with Sprouty4 overexpression (p < 0.001) as well as MEK inhibition (p < 0.05). These observations were substantiated by Boyden chamber assays where significant decreases in invasion were observed by both the MCF10.CA1d/S1 and MCF10.CA1d/S2 lines (p < 0.01) compared to MCF10.CA1d (see Fig 9).

**Fig 10:**
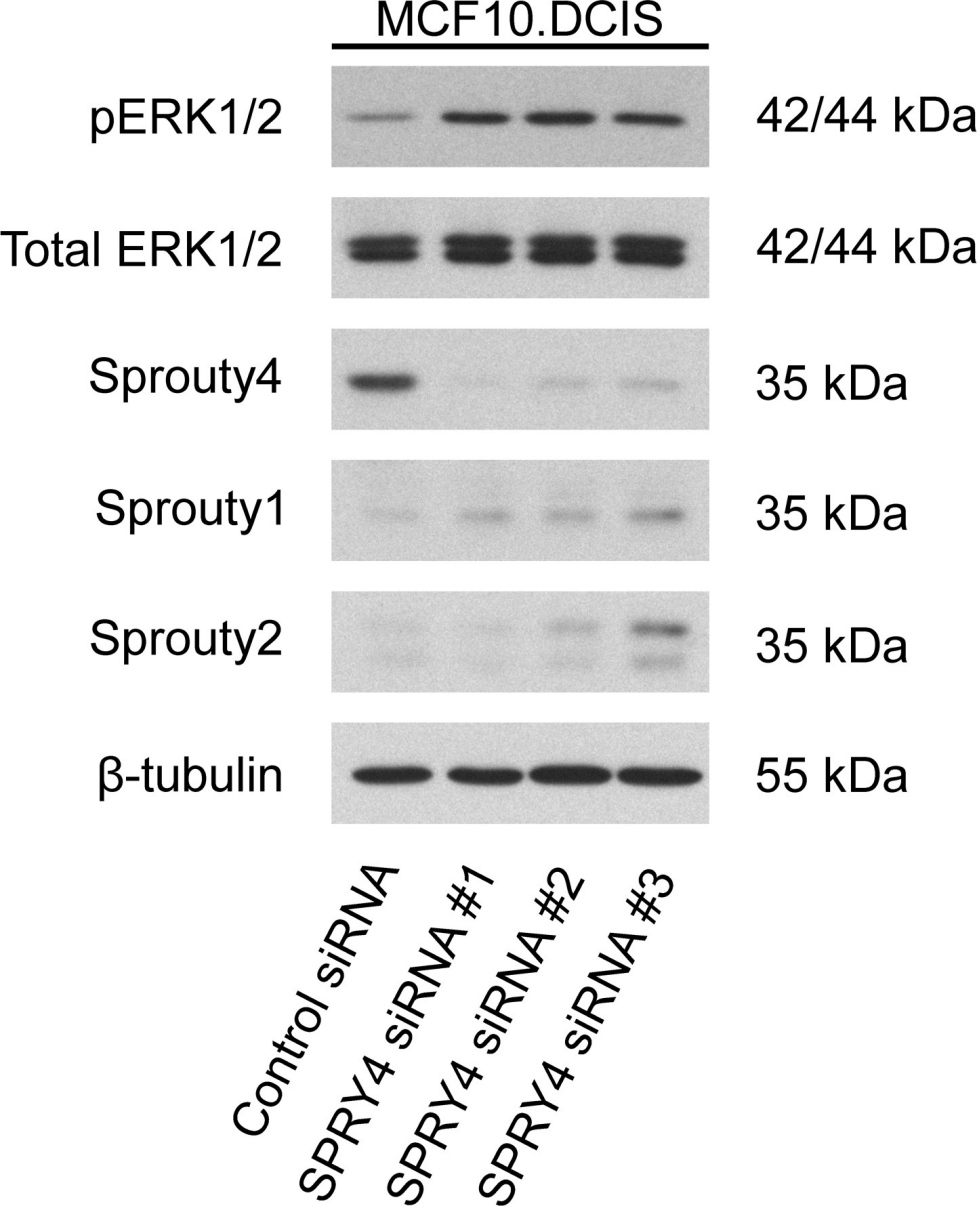
Transient knockdown of Sprouty4 in MCF10.DCIS cells enhances ERK/MAPK signaling. MCF10.DCIS cells were grown in 2D culture, then transfected with either a non-targeting control siRNA or one of three non-overlapping siRNA constructs targeting SPRY4 for 48 hours. Membranes were immunoblotted for phosphorylated ERK1/2 (pERK1/2), total ERK1/2, Sprouty4, Sprouty1, Sprouty2, and β-tubulin. Expression levels are representative of three independent experiments.

**Fig 11:**
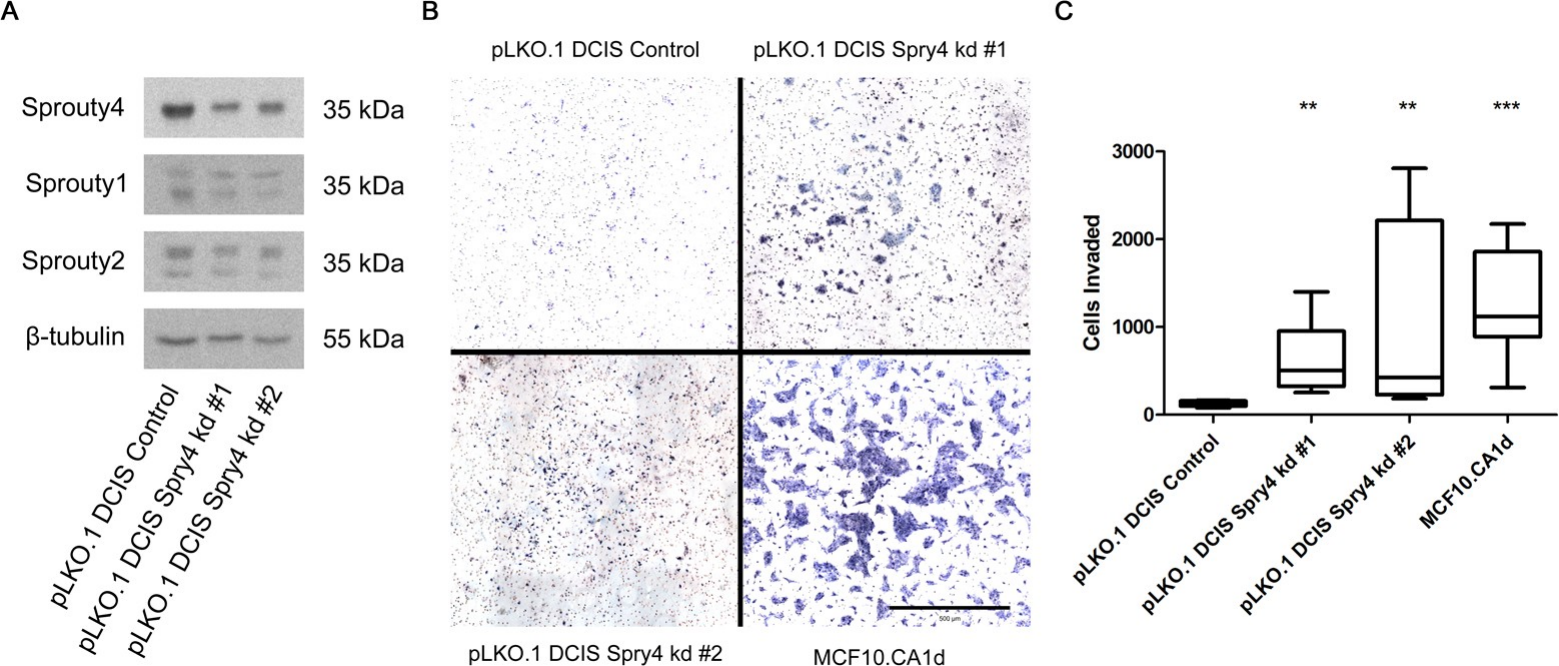
Stable knockdown of Sprouty4 in MCF10.DCIS cells promotes invasion. (A) MCF10.DCIS cells were infected with lentivirus containing either vector control or Sprouty4-targeting shRNA constructs. Membranes were immunoblotted for Sprouty4, Sprouty1, Sprouty2, and β-tubulin. (B) pLKO.1 DCIS control, pLKO.1 DCIS Sprouty4 knockdown, and MCF10.CA1d cells were then serum-starved overnight and allowed to invade though 1.5 mg/mL of matrix for 24 hours via Boyden chamber assay. Representative images of cellular invasion (scale bar = 500 μm). (C) Invasion was quantified and plotted using GraphPad Prism. A Kruskal-Wallis test employing Dunn’s test of multiple comparisons showed significant differences in invasion when comparing Sprouty4 knockdown or MCF10.CA1d cell lines to control (α = 0.01, 99% CI; ** p < 0.01, *** p < 0.001). Whiskers represent the minimum and maximum values and bars represent the median. Data were collected from three independent experiments.

As a complementary approach to confirm that Sprouty4 and the ERK/MAPK pathway regulate pericellular proteolysis, we tested the effects of reducing Sprouty4 expression. First, we transiently knocked down Sprouty4 using three non-overlapping siRNA constructs. As shown in Fig 10, knockdown of Sprouty4 in MCF10.DCIS cells resulted in increased ERK1/2 phosphorylation. We then generated two independent, stable shRNA knockdowns of Sprouty4 in MCF10.DCIS (see Fig 11A). In Boyden chamber invasion assays these stable knockdown lines, as well as MCF10.CA1d cells which acted as a positive control, showed significantly higher invasion (p < 0.01 and p < 0.001, respectively) compared to DCIS control (see Fig 11B and 11C). Taken together, these data strongly implicate Sprouty4 as a regulator of DCIS cell invasion.

## Discussion

Sprouty was initially characterized as an antagonist of FGF signaling during *Drosophila* development [23]. Since then, a sizable body of literature has established that this regulatory protein and its mammalian homologs (Sproutys1-4) primarily act to suppress growth factor-induced ERK signaling [26]. In breast cancer, the overactivation of ERK/MAPK signaling is well documented [36, 63]. Our staining of normal, DCIS, and IDC breast tissues corroborates this observation in that significantly more ERK/MAPK phosphorylation is present in IDC tissues compared to normal breast samples. Importantly, we observed a similar pattern when comparing the levels of ERK/MAPK phosphorylation between invasive and DCIS tissues. This is likely the result of growth suppressors or other regulatory measures actively working to keep these premalignant lesions in check. The nature of this regulation is still somewhat unclear. Therefore, identifying molecular alterations that consistently differ between *in situ* and invasive disease remains a priority [3, 4, 64, 65].

Loss of Sprouty4 has been previously reported for a number of different cancers. In prostate, colorectal, and hepatocellular carcinomas the *SPRY4* transcript has been shown to be lower in comparison to the respective normal tissues [38, 66, 67]. Decreased expression has also been noted at the protein level in endometrial adenocarcinoma [68]. An oncogenic role for microRNA-181 was also reported in breast cancer, in part by targeting the 3’ untranslated region of *SPRY4* [69]. To the best of our knowledge, the present study is the first to suggest a role for Sprouty4 in limiting premalignant breast lesions from transitioning to invasive disease. Analysis of IDC vs. normal breast tissue using the Oncomine database showed that the *SPRY4* transcript is significantly underexpressed in IDC samples in both the TCGA (p = 1.06x10^-5^) and Curtis Breast (p = 8.78x10^-10^) datasets. These data differ from a previously published meta-analysis conducted by Faratian *et al*. in which no significant differences in the *SPRY4* transcript were found between invasive and normal breast tissues [70]. One possible explanation for this difference is the quantity of tumor samples interrogated (42 in their study vs. 389 or 1556 for the TCGA or Curtis datasets, respectively). In addition, we validated these datasets in patient tissues by immunohistochemistry, demonstrating that Sprouty4 expression is significantly lower in invasive samples relative to DCIS and normal breast. This pattern prevails when IDCs are pooled as well as when they are separated into hormone positive, HER2 amplified or triple-negative categories, indicating that loss of Sprouty4 is not an event specifically tied to a single clinical subtype of breast cancer. This loss of Sprouty4 expression between DCIS and IDC was reproducible using 3D culture techniques and the MCF10 series to model breast cancer progression. Knockdown of Sprouty4 in MCF10.DCIS cells substantially increased ERK/MAPK signaling whereas overexpression in invasive MCF10.CA1d cells decreased ERK/MAPK signaling. While breast cancer-specific mutations or deletions have not been described, mutations of unknown significance are documented in the COSMIC database (https://cancer.sanger.ac.uk/cosmic/search?q=spry4) and may be worthy of future study.

The RTK/Ras/ERK pathway continues to garner a high level of scientific interest [71–73]. This is likely because it plays a critical role in a number of cellular functions (e.g., proliferation, differentiation, invasion) where the activity of key pathway members is tightly regulated spatially and temporally [74]. One way that this regulation is frequently circumvented in cancer is through gain-of-function mutations in three of the central players: RTKs (e.g., EGFR), Ras, and Raf [75].

While present as mutated, driving oncogenes in certain tumors there is also significant evidence that wild-type Ras isoforms contribute to the malignant phenotype [76]. For example, in breast carcinoma there is a common theme of Ras pathway activation through multiple mechanisms, including neurofibromin loss and overexpressed growth factor receptors, while Ras mutations themselves are rarely found [77, 78]. Due to difficulties in directly targeting Ras or its association with membranes [reviewed in [79]], most efforts have shifted to developing targeted inhibitors of downstream proteins driven by activated Ras [80, 81]. This strategy has been vindicated by the FDA’s approval of three MEK1/2 inhibitors: trametinib in 2013, cobimetinib in 2015, and MEK162/binimetinib in 2018 (in combination with the Raf inhibitors dabrafenib, vemurafenib, and encorafenib, respectively) for the treatment of advanced melanoma with a BRAF V600E or V600K mutation [82–85]. In our study, pharmacological inhibition of MEK1/2 in MCF10.CA1d cells reduced ERK/MAPK signaling as expected, and also reduced Sprouty4 expression. This indicates that with less active ERK1/2 signaling to regulate there is a decreased need for Sprouty4 expression. Collectively, these data, the previously mentioned Sprouty4 knockdown and overexpression experiments, as well as the tissue staining for ERK1/2 phosphorylation demonstrate Sprouty4’s ability to regulate and respond to ERK/MAPK signaling in premalignant breast tissue and suggest that loss of its regulation in IDC may be important for the transition to invasive disease.

Although this work does not directly address binding or where in the pathway Sprouty4 acts, two potentially useful pieces of information can be gleaned from our studies. The first is that Sprouty4 operates upstream of MEK1/2. This can be inferred from the reduction of phosphorylated MEK when Sprouty4 is overexpressed in MCF10.CA1d cells as it contrasts with the substantial increase seen when MCF10.CA1d cells are treated with nanomolar concentrations of the MEK inhibitors PD184352 or MEK162. Pharmacological inhibition of MEK leads to the depletion of ERK1/2 signaling and prevents the feedback signaling normally responsible for MEK dephosphorylation. As this pattern is not present with Sprouty4 overexpression, one may reasonably assume that this protein performs its regulatory function further upstream in the pathway. These data are consistent with literature showing that Sprouty4 acts at the level of Ras or with Raf proteins [24, 57, 58].

The second, more intriguing piece of the puzzle is tangential to prior work from our laboratory showing increased levels of activated H-Ras in MCF10.DCIS cells [86]. This detail is relevant to the present study because knocking down Sprouty4 in MCF10.DCIS cells dramatically increases ERK/MAPK signaling. Such an increase in the presence of a constitutively active Ras suggests Sprouty4 may perform its regulatory action downstream of Ras, potentially at the level of Raf as previously reported in melanoma [58]. Alternatively, Sprouty4 may interact with Ras-GAP as described in *Drosophila* [25]. There are caveats to these assumptions, however. The first is that the role that transduced H-Ras plays in the MCF10 series is unclear, as it has been reported to be insufficient for producing the premalignant stem cell phenotype [49]. In addition, all cell lines in the MCF10 series are reliant on growth factor supplementation suggesting that the proliferation of variants harboring the T24-H-Ras, such as MCF10.DCIS, is not driven entirely by this oncogene. Further testing of active Ras and Raf protein levels would be necessary to validate these potential interactions, ideally using additional DCIS or breast cancer cell lines which do not harbor Ras mutations. If accurate, this method of regulating ERK/MAPK signaling would add to the list of ways Sprouty4 behaves differently from Sprouty1 and Sprouty2 and would underscore Sprouty4’s biological importance to breast cancer [87].

Overexpressing Sprouty4 in invasive MCF10.CA1d cells, in addition to suppressing ERK1/2 phosphorylation, leads to changes in Slug, E-cadherin, and the actin cytoskeleton. A reduction in levels of Slug is likely explained by its role as a transcription factor downstream of the ERK/MAPK cascade [55, 56]. Prolonged inhibition (i.e., 48 hours) of the ERK/MAPK pathway in breast cancer cells has been shown to decrease Slug expression and inhibit cell migration [54]. We also observe minimal increases in E-cadherin protein expression upon Sprouty4 overexpression or MEK inhibition, and suspect this is related to the reduction in Slug levels due to Slug’s ability to repress E-cadherin in breast carcinoma cell lines [59]. Our previous work showing that MAPK inhibition was able to contribute to the restoration of E-cadherin cell-cell junctions prompted inspection of E-cadherin localization [86]. We demonstrated that in MCF10.CA1d cells overexpressing Sprouty4 or treated with a MEK inhibitor, E-cadherin localizes to the cell surface and the actin cytoskeleton is dramatically reorganized to resemble an epithelial morphology. This contrasts with prior work by Tsumura *et al*. in which Sprouty4 was reported to regulate the actin cytoskeleton independently of the ERK/MAPK pathway [88]. Possible explanations for this difference include differences in cell lines used as well as the duration (20 minutes vs. 48 hours in our study) and concentration (20 μM vs. 100 nM in our study) at which MEK inhibitors were applied.

As previously mentioned, identifying molecules whose expression consistently differs between *in situ* and invasive disease remains an important goal. Attempts to address this objective have come in the form of gene expression panels like the Oncotype DX DCIS Score, transcriptomic analyses such as the one performed by our laboratory which led to this study, as well as the proposition of candidate biomarkers [21, 65, 89]. Prognostic biomarkers are used to indicate the likely course of disease in an untreated individual [90, 91]. They mainly benefit lower risk patients because the information gleaned is then used to select the most appropriate type of adjuvant treatment (e.g., the addition or absence of radiotherapy after breast conservation surgery) [90]. Unfortunately, due to limited tissue most DCIS biomarker studies are underpowered or diluted by the inclusion of samples with an invasive component [65]. Differences in IHC scoring methods also have the potential to limit the impact of these studies. While our IHC staining suggests that Sprouty4 is not a suitable biomarker candidate, our results collectively support its role in limiting the transition to invasive disease.

In conclusion, we have identified Sprouty4 as an important regulator of ERK/MAPK signaling in DCIS, thus limiting the progression of these premalignant breast lesions. Through *in silico* analyses, IHC staining of human DCIS and IDC patient tissues, as well as the use of an *in vitro* 3D model of disease progression we found that Sprouty4 expression was substantially reduced with progression to IDC. In the absence of Sprouty4 regulation, ERK/MAPK phosphorylation increased, both in human tissue and when artificially silenced in cells. Reduction of Sprouty4 also promoted invasiveness while the opposite was true of overexpression. Images of invasive cells overexpressing Sprouty4 revealed data consistent with phenotypic reversion, such as remodeling of the actin cytoskeleton, relocation of E-cadherin back to the cell surface (suggesting the restoration of adherens junctions), and partial formation of acinar-like structures. The use of MEK inhibitors confirmed that these effects were ultimately driven by reductions in ERK/MAPK signaling, as pharmacological inhibition also produced a phenotype similar to Sprouty4 overexpression.

## Supporting information

S1 Fig SPRY4 transcript decreases for IDCs compared to normal tissue in the TCGA Breast dataset. Data mining of the TCGA Breast dataset revealed a 1.53-fold decrease in SPRY4 mRNA expression in IDCs (n = 389) compared to non-cancerous breast tissue (n = 61). The Student’s t-test was employed for statistical analysis; p = 1.05x10^−5^. The Oncomine Platform (Thermo Fisher, Ann Arbor, MI) was used for analysis and visualization.
(TIF)

S2 Fig SPRY4 transcript decreases for IDCs compared to normal tissue in the Curtis Breast dataset. Data mining of the Curtis Breast dataset revealed a 1.20-fold decrease in SPRY4 mRNA expression in IDCs (n = 1556) compared to non-cancerous breast tissue (n = 144). The Student’s t-test was employed for statistical analysis; p = 8.78x10^−10^. The Oncomine Platform (Thermo Fisher, Ann Arbor, MI) was used for analysis and visualization.
(TIF)

S3 Fig Staining for Sprouty4 is reduced and for ERK1/2 phosphorylation is increased in human IDCs relative to normal and DCIS tissue. Tissue microarrays containing samples of human normal tumor adjacent breast, n = 24; DCIS, n = 45; and IDC, n = 169; were processed for IHC using optimized protocols and antibodies for Sprouty4 and phosphorylated ERK1/2 (pERK1/2). Sprouty4 and pERK1/2 expression levels depicted for each tissue represent median staining values; size bar = 100 μm. While recommended for IHC by the manufacturer, the Sprouty4 antibody produced a consistent nuclear signal across all samples in addition to detecting changes in cytosolic protein levels. Further validation of the probe through targeted knockdown experiments confirmed it was indeed capable of selectively detecting Sprouty4 (see Fig 10).
(TIF)

S4 Fig Pharmacological inhibition of ERK/MAPK signaling leads to changes in cellular organization. Differential interference contrast images of control and MEK inhibited MCF10.CA1d cells grown in 2D were captured using a Zeiss Cell Observer spinning disk confocal microscope with a 5x objective; size bar = 500 μm. The ERK/MAPK pathway was targeted for 48 hours using three separate MEK1/2 inhibitors: U0126 (10 μM), PD184352/CI-1040 (100 nM), and MEK162/binimetinib (100 nM). DMSO and U0124 (an inactive form of U0126) served as negative controls. Changes in cellular organization were observed in each case of MEK inhibition in sharp contrast with control cells. Images are representative of three independent experiments.
(TIF)

S1 Video 360° view of E-cadherin (red), filamentous actin (green), and nuclei staining (blue) in 3D structures formed by MCF10.DCIS cells. Images were obtained on a Zeiss LSM 780 confocal microscope and converted to a movie file using Volocity software. This movie complements the equatorial snapshot shown in Fig 6.
(MP4)

S2 Video **360° view of E-cadherin (red), filamentous actin (green), and nuclei staining (blue) in 3D structures formed by DMSO-treated MCF10.CA1d cells.** Images were obtained on a Zeiss LSM 780 confocal microscope and converted to a movie file using Volocity software. This movie complements the equatorial snapshot shown in Fig 6.
(MP4)

S3 Video **360° view of E-cadherin (red), filamentous actin (green), and nuclei staining (blue) in 3D structures formed by MCF10.CA1d/S1 cells.** Images were obtained on a Zeiss LSM 780 confocal microscope and converted to a movie file using Volocity software. This movie complements the equatorial snapshot shown in Fig 6.
(MP4)

S4 Video **360° view of E-cadherin (red), filamentous actin (green), and nuclei staining (blue) in 3D structures formed by MCF10.CA1d/S2 cells.** Images were obtained on a Zeiss LSM 780 confocal microscope and converted to a movie file using Volocity software. This movie complements the equatorial snapshot shown in Fig 6.
(MP4)

S5 Video **360° view of E-cadherin (red), filamentous actin (green), and nuclei staining (blue) in 3D structures formed by MEK162-treated MCF10.CA1d cells.** Images were obtained on a Zeiss LSM 780 confocal microscope and converted to a movie file using Volocity software. This movie complements the equatorial snapshot shown in Fig 6.
(MP4)

S1 Raw images (PDF)

S1 File (DOCX)
