# Supplementary figures and images for: Sprouty4 negatively regulates ERK/MAPK signaling and the transition from in situ to invasive breast ductal carcinoma
Source: PLoS One. 2021 May 28;16(5):e0252314. doi: 10.1371/journal.pone.0252314 (PMC8162601; doi:10.1371/journal.pone.0252314)

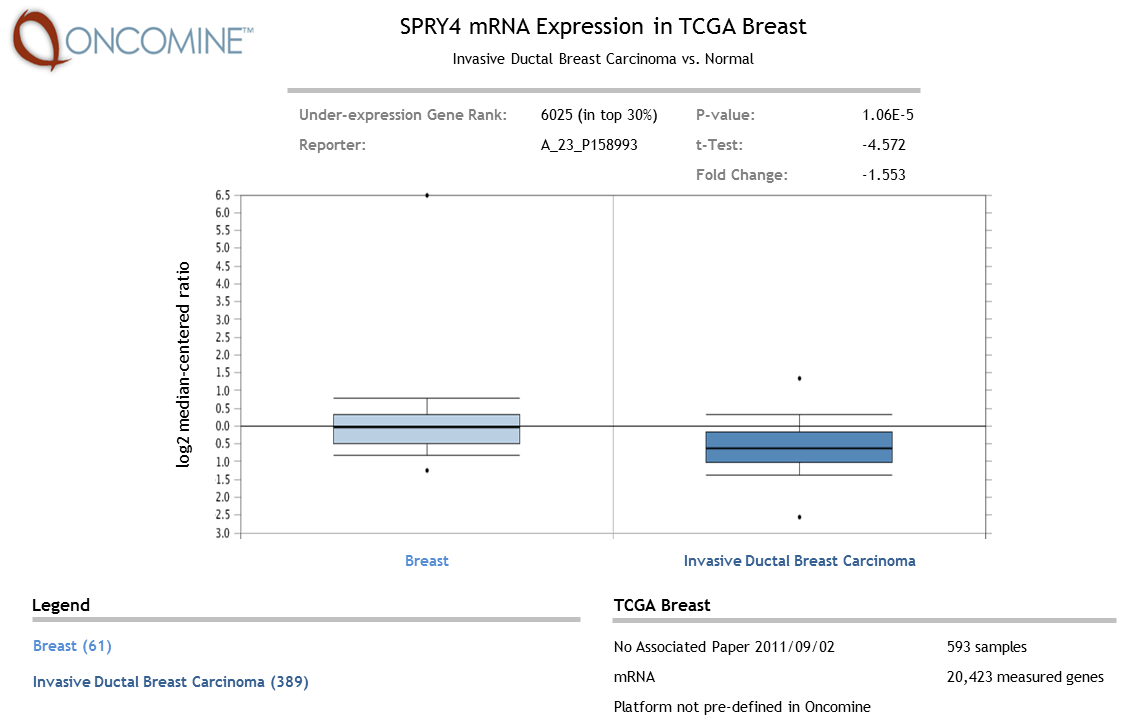

Supplement: S1 Fig — Data mining of the TCGA Breast dataset revealed a 1.53-fold decrease in SPRY4 mRNA expression in IDCs (n = 389) compared to non-cancerous breast tissue (n = 61). The Student’s t-test was employed for statistical analysis; p = 1.05x10^−5^. The Oncomine Platform (Thermo Fisher, Ann Arbor, MI) was used for analysis and visualization. (TIF) [file pone.0252314.s001.tif]

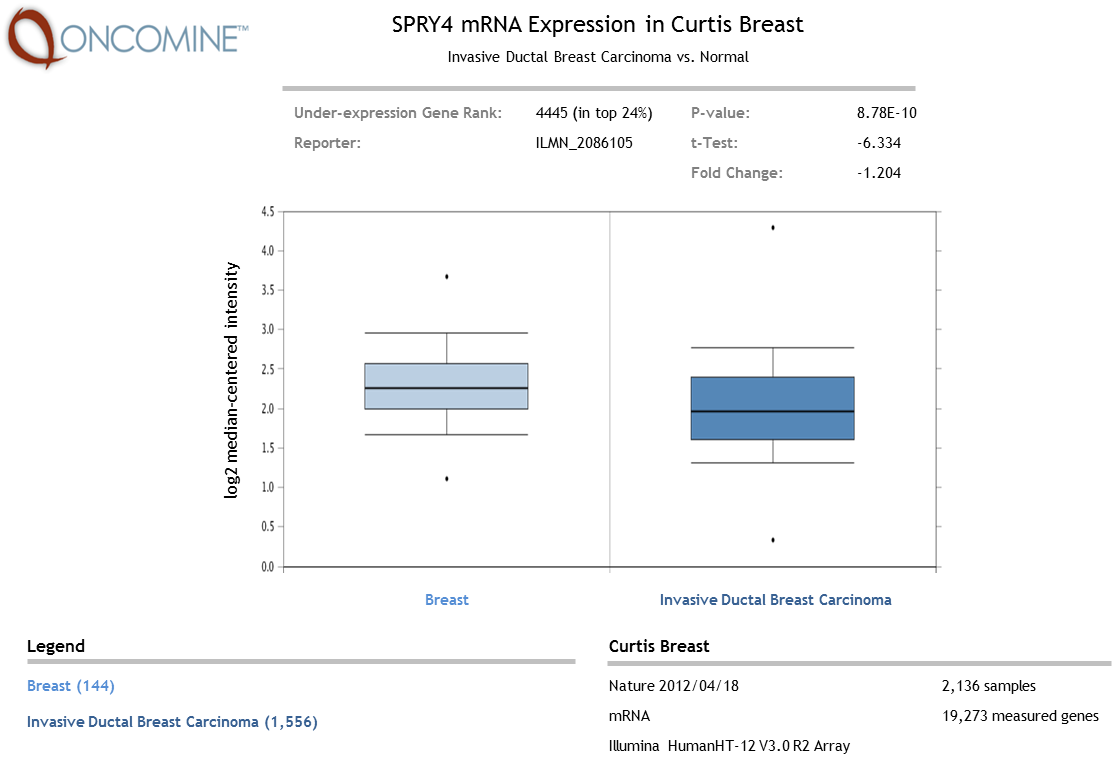

Supplement: S2 Fig — Data mining of the Curtis Breast dataset revealed a 1.20-fold decrease in SPRY4 mRNA expression in IDCs (n = 1556) compared to non-cancerous breast tissue (n = 144). The Student’s t-test was employed for statistical analysis; p = 8.78x10^−10^. The Oncomine Platform (Thermo Fisher, Ann Arbor, MI) was used for analysis and visualization. (TIF) [file pone.0252314.s002.tif]

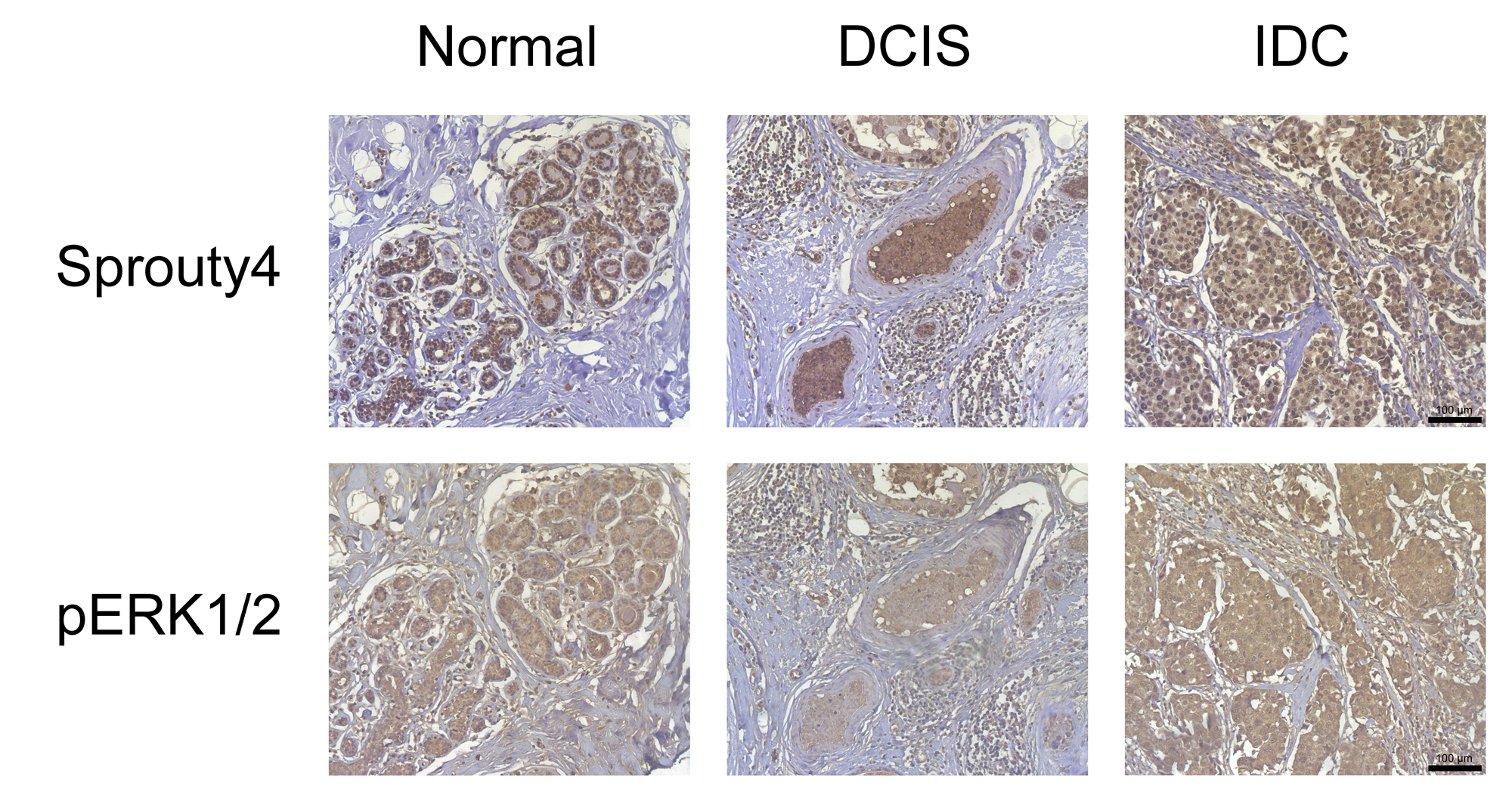

Supplement: S3 Fig — Tissue microarrays containing samples of human normal tumor adjacent breast, n = 24; DCIS, n = 45; and IDC, n = 169; were processed for IHC using optimized protocols and antibodies for Sprouty4 and phosphorylated ERK1/2 (pERK1/2). Sprouty4 and pERK1/2 expression levels depicted for each tissue represent median staining values; size bar = 100 μm. While recommended for IHC by the manufacturer, the Sprouty4 antibody produced a consistent nuclear signal across all samples in addition to detecting changes in cytosolic protein levels. Further validation of the probe through targeted knockdown experiments confirmed it was indeed capable of selectively detecting Sprouty4 (see Fig 10). (TIF) [file pone.0252314.s003.tif]

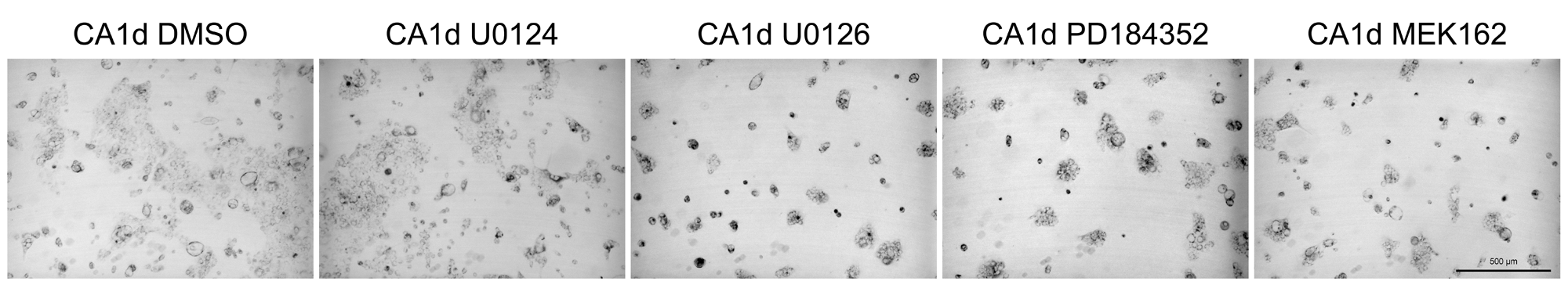

Supplement: S4 Fig — Differential interference contrast images of control and MEK inhibited MCF10.CA1d cells grown in 2D were captured using a Zeiss Cell Observer spinning disk confocal microscope with a 5x objective; size bar = 500 μm. The ERK/MAPK pathway was targeted for 48 hours using three separate MEK1/2 inhibitors: U0126 (10 μM), PD184352/CI-1040 (100 nM), and MEK162/binimetinib (100 nM). DMSO and U0124 (an inactive form of U0126) served as negative controls. Changes in cellular organization were observed in each case of MEK inhibition in sharp contrast with control cells. Images are representative of three independent experiments. (TIF) [file pone.0252314.s004.tif]
