# Supplementary material for: Sprouty4 negatively regulates ERK/MAPK signaling and the transition from in situ to invasive breast ductal carcinoma
Source: PLoS One. 2021 May 28;16(5):e0252314. doi: 10.1371/journal.pone.0252314 (PMC8162601; doi:10.1371/journal.pone.0252314)

Figure: 2
Method: chemiluminescence to film

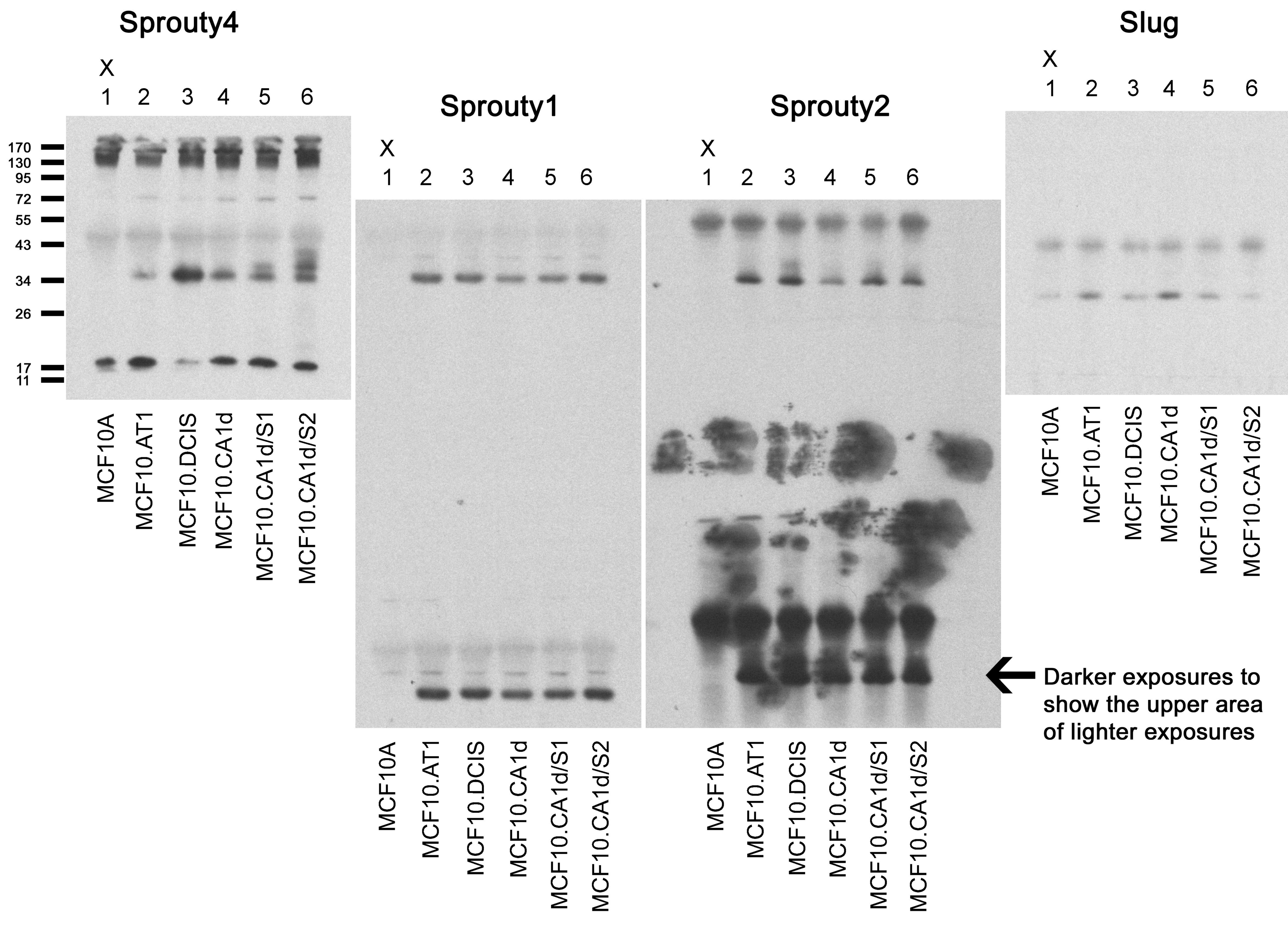

Figure: 3
Method: chemiluminescence to film

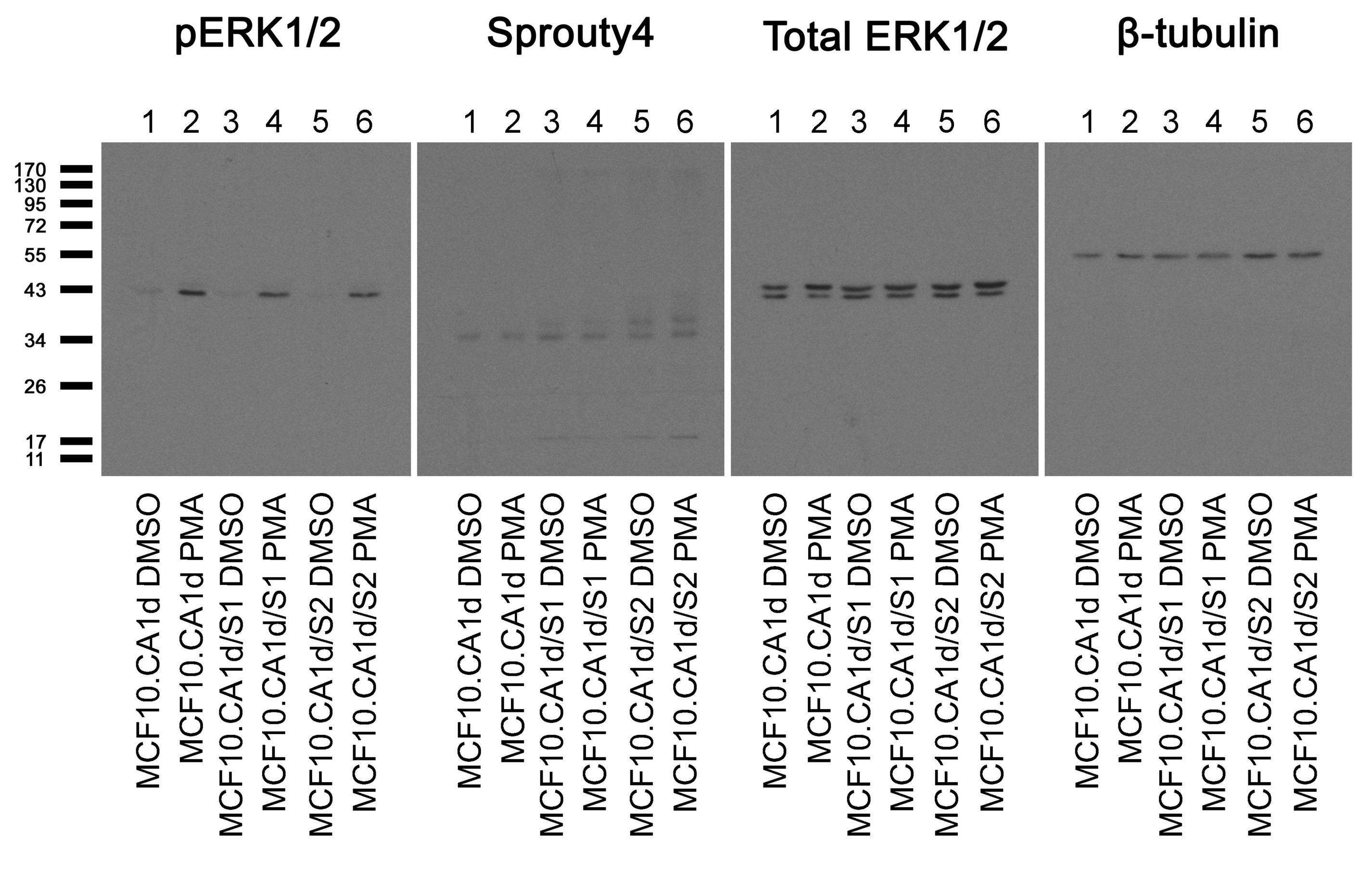

Figure: 4
Method: chemiluminescence to film

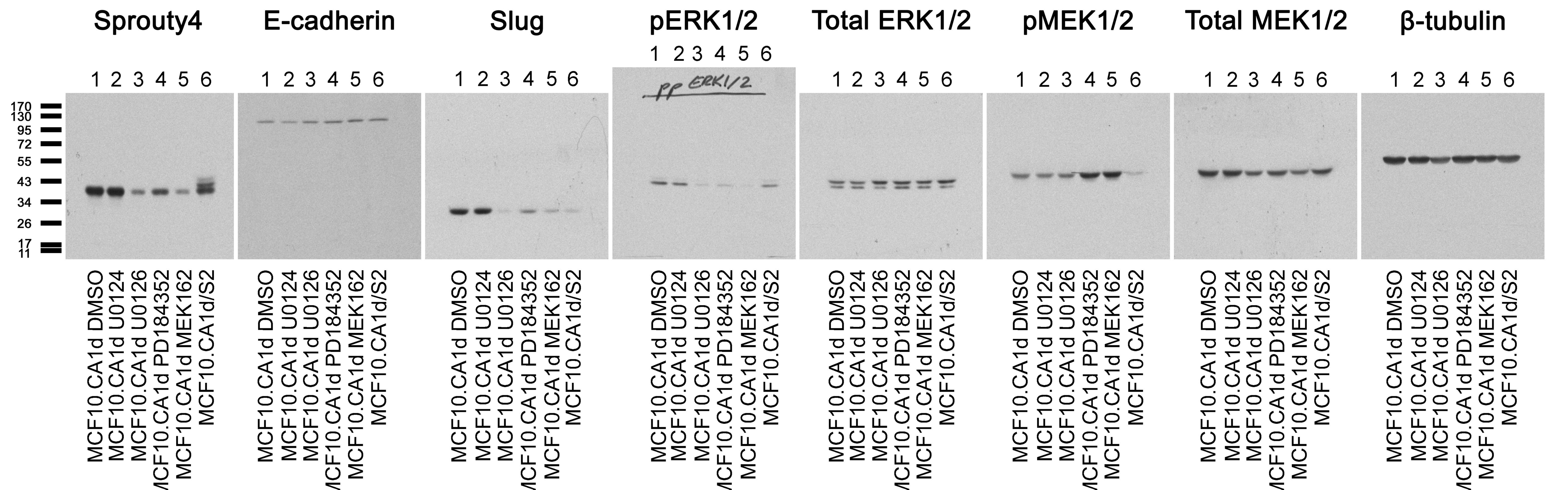

Figure: 10
Method: chemiluminescence to film

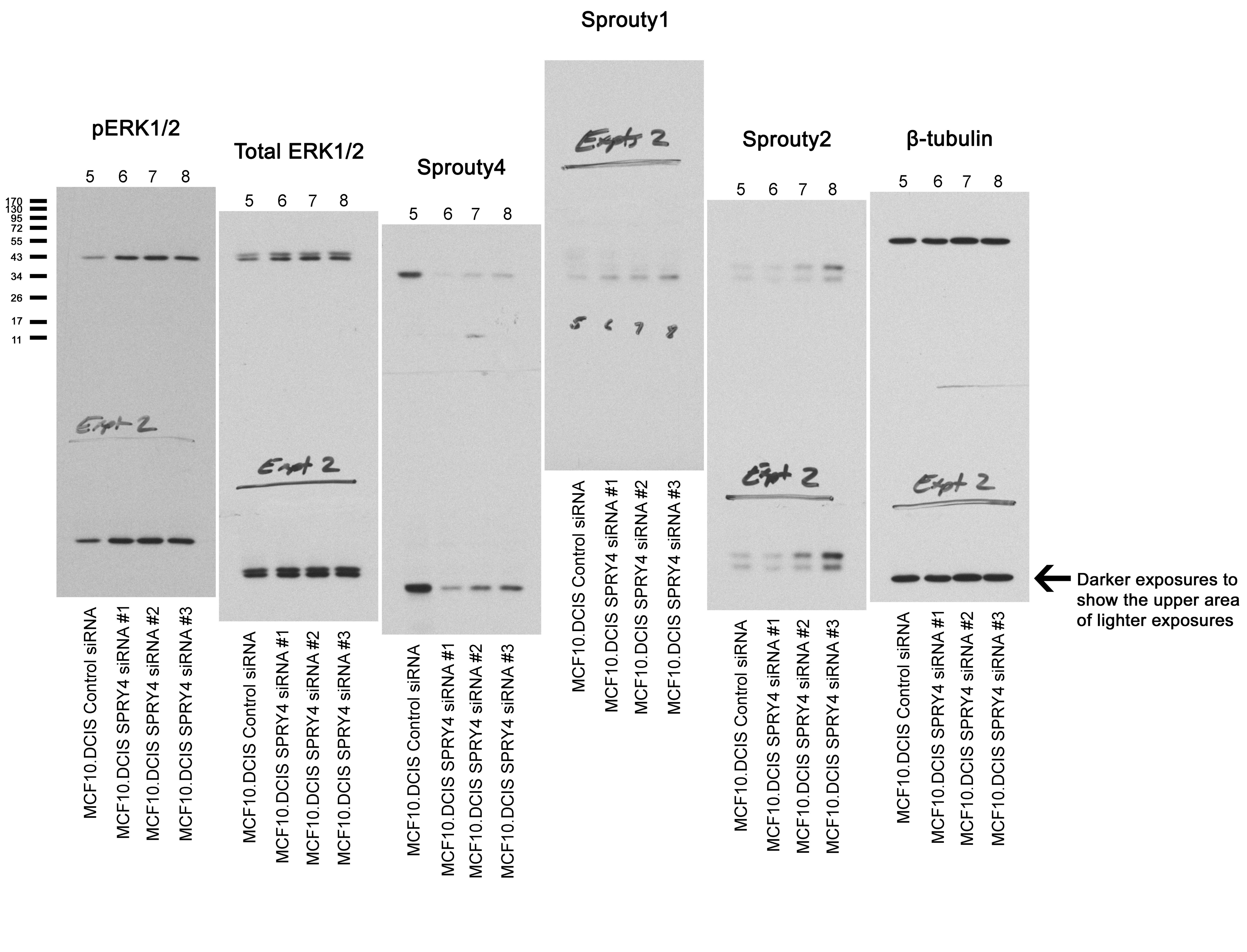

Figure: 11
Method: chemiluminescence to film

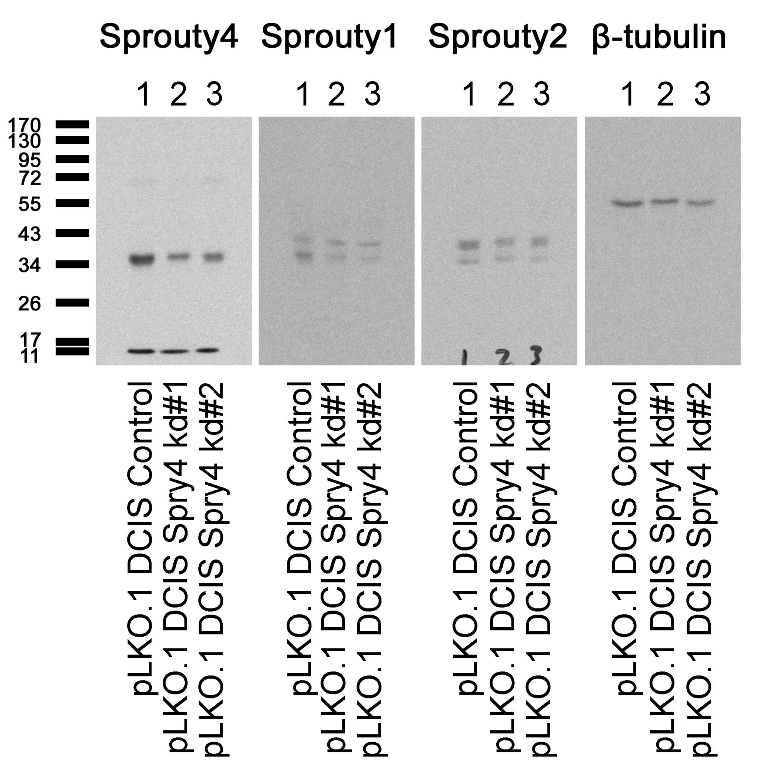

Supplement: S1 Raw images — (PDF) [file pone.0252314.s010.pdf]
