# Supplementary material for: Sprouty4 negatively regulates ERK/MAPK signaling and the transition from in situ to invasive breast ductal carcinoma
Source: PLoS One. 2021 May 28;16(5):e0252314. doi: 10.1371/journal.pone.0252314 (PMC8162601; doi:10.1371/journal.pone.0252314)
Supplement: S1 File — (DOCX) [file pone.0252314.s011.docx]

**Supporting Methods**

***In Silico* Analyses**

The Oncomine database (Oncomine v4.5: 729 datasets, 91,866 samples) was used for the analysis of non-cancerous breast tissue *vs.* invasive ductal breast carcinoma (filters: Invasive Ductal Breast Carcinoma vs. Normal Analysis; *SPRY4*). In both cases, the data were ordered by ‘under-expression’ of the target gene, and the threshold was adjusted to *P*-value < 1x10^-4^, fold change 2, gene rank top 10%.
